# Respiratory syncytial virus inhibits type I interferon signaling to maintain HLA-DM expression in CD1c^+^ dendritic cells

**DOI:** 10.1016/j.isci.2026.116736

**Published:** 2026-07-10

**Authors:** Weiyee Ong, Richard Anthony Hopkins, Enjun Yang, Novita, Najwa Talib, Bijin Au, John Edward Connolly

**Affiliations:** 1 Institute of Molecular and Cell Biology (IMCB), Agency for Science, Technology and Research (A∗STAR), Singapore, Singapore; 2 Department of Microbiology and Immunology, Yong Loo Lin School of Medicine, National University of Singapore, Singapore, Singapore; 3 Institute of Biomedical Studies, Baylor University, Waco, TX 76712, USA

**Keywords:** respiratory syncytial virus, RSV, dendritic cell, DC, HLA-DM, type I interferon pathway, TBK1, antigen presentation, immune evasion

## Abstract

Respiratory syncytial virus (RSV) infection often elicits ineffective long-term immune responses due to inefficient immune priming, complicating disease management and vaccine development. Dendritic cells (DCs) are central regulators of antiviral immunity and antigen presentation; yet, the direct impact of RSV on these pathways remains poorly understood. In this study, we identify sustained HLA-DM expression as a unique hallmark of RSV infected CD1c^+^ DCs, a phenotype not observed following influenza infection or poly(I:C) stimulation. Using single cell RNA sequencing, pharmacological inhibition, and complementary controls, we demonstrate that TBK1 dependent Type I Interferon signaling is a key regulator of HLA-DM expression during DC maturation. Co-culture experiments further suggest that HLA-DM-high DCs influence CD4^+^ T cell differentiation, supporting a model in which sustained HLA-DM expression reshapes antigen presentation and downstream adaptive immunity. Together, these findings uncover a previously unknown link between innate antiviral signaling and antigen presentation machinery in human DCs.

## Highlights

• RSV sustains HLA-DM expression in RSV infected CD1c+ dendritic cells
• RSV disrupts IRF3 dependent Type I interferon transcription
• TBK1 inhibition recapitulates RSV induced HLA-DM upregulation
• HLA-DMhi dendritic cells induce CD4^+^ T cell states linked to poor outcomes

## Graphical abstract

**Figure:**
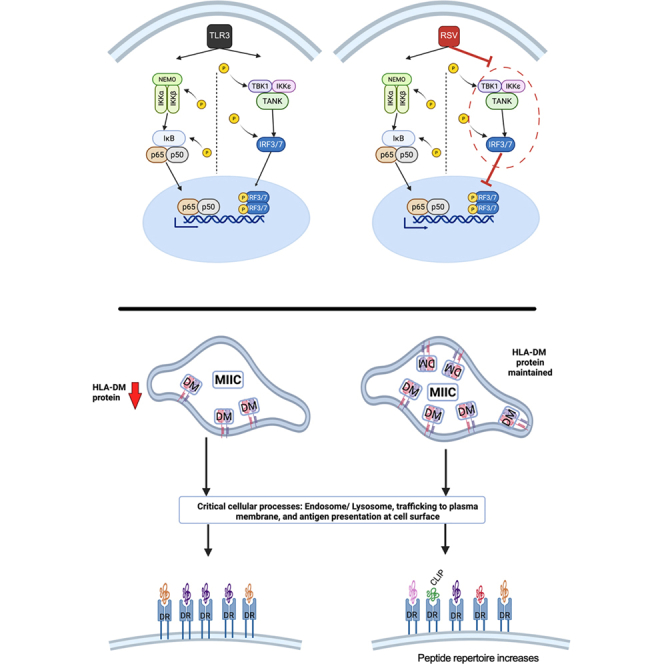

## Teaser

Immunology; Virology; Cell biology

## Introduction

Respiratory syncytial virus (RSV), from the *Paramyxoviridae* family, is a highly infectious negative-sense RNA virus. RSV is prevalent in over 90% of pediatric populations before the age of two,^1^ with approximately 30 million children globally below age five exhibiting lower respiratory tract infections (LRTI), with a tenth of them requiring hospitalization.^1^^,^^2^ RSV infection induces a Th2 driven, pro-inflammatory immune response, which hinders the development of immunological memory, resulting in recurrent reinfections throughout life.^2^^,^^3^^,^^4^ Most recent RSV vaccine strategies e.g., nirsevimab have reduced overall hospitalization rates of infected patients by up to 80%, however, they struggle to maintain antibody half-life to protect against reinfection.^5^ Moreover, insufficient attention is given to stimulation of other leukocytes involved in long-term immune responses; hence, RSV remains a long-standing and significant health issue worldwide.^6^ Increasing evidence shows that RSV negatively regulates dendritic cell (DC) function by affecting innate and adaptive anti-viral immune responses, inducing inefficient DC maturation, poor antigen presentation and inhibited antiviral type I interferon (IFN) signaling. However, the molecular mechanisms and pathways contributing to DC dysfunction in RSV infection remain elusive.^7^^,^^8^^,^^9^^,^^10^ Further research in this area remains a priority for unlocking potent vaccination strategies.

The induction of adaptive immune responses by DCs involves complex processes, with antigen presentation playing a central role. This process involves extracellular ligand-receptor interactions, in a process termed antigen presentation.^8^ Antigens from the pathogen are processed into peptides and loaded onto major histocompatibility molecules (MHCII) (HLA- DR, -DP, and -DQ) which then traffic to DC surfaces for downstream engagement with T cells.^11^ Antigen peptide selection is critical for effective T cell priming because the strength of T cell receptor-peptide-MHCII (TCR-*p*-MHCII) interactions can affect T cell responsiveness, differentiation and longevity in circulation.^12^^,^^13^ Therefore, strong affinity peptide-MHC conjugates stabilize the avidity of TCR-P-MHCII during interaction, expanding more high affinity TCR clones capable of recognizing and mounting stronger effector responses.^12^^,^^14^ Peptide-MHC complex formation is dependent on (1) MHCII genetic polymorphisms, which cause residue variation at the peptide binding groove^11^ and (2) HLA-DM expression. HLA-DM is a peptide loading facilitator that stabilizes the MHCII binding groove during the displacement of weak affinity self-peptide CLIP for a higher affinity antigenic peptide.^11^ Upon DC activation, HLA-DM expression decreases, enabling peptide loading.^15^^,^^16^^,^^17^ Conversely, higher levels of HLA-DM results in the expansion of the DC peptide repertoire, reducing DC antigen specificity and triggering promiscuous effector or memory T cell expansion.^15^^,^^18^^,^^19^ Hence, to control aberrant HLA-DM function, DCs express HLA-due to inhibit peptide loading by competitively binding with HLA-DM and inhibiting MHCII-HLA-DM interactions.^19^^,^^20^^,^^21^ While the mechanisms of antigen presentation are crucial for initiating adaptive immune responses, other factors, such as cytokine signaling, also play a vital role in shaping the overall immune response to viral infections.

One key group of cytokines in this process are the type I IFNs, which are critical anti-viral mediators crucial in shaping the adaptive immune response against viruses. DCs secrete the greatest amount of IFNs, which act in an autocrine fashion to enhance activation signaling.^22^^,^^23^ RSV’s non-structural (NS) proteins are known to directly associate and inhibit multiple molecular targets within the type I IFN pathway during infection.^24^^,^^25^^,^^26^^,^^27^^,^^28^^,^^29^ While RSV’s inhibition of the type I IFN pathway is established, the specific mechanisms by which this inhibition affects DC antigen presentation remain unclear. In particular, the role of impaired type I IFN signaling in altering DC antigen presentation during RSV infection is not well understood. Pursuant to this, we characterized the effect of acute RSV infection on CD1c^+^ DCs, the major subset of human blood DCs involved in antiviral adaptive responses.

We demonstrated that despite having similar surface activation marker profiles as DCs activated with agonists, only RSV infected DCs maintained MIIC resident HLA-DM. scRNA sequencing and pharmacological inhibition of the downstream signaling molecule TBK1 confirmed type I IFN signaling as a crucial regulator of HLA-DM expression, independent of JAK/STAT signaling. To illustrate the impact of varying HLA-DM levels on subsequent T cell responses, we employed pharmacological inhibitors to generate HLA-DM^hi^ and HLA-DM^lo^ DCs. These DCs were co-cultured with autologous antigen specific T (VST) cells, focusing on proliferation and cell surface marker expression. Co-culture data suggest that high HLA-DM expression is associated with altered T cell differentiation phenotype, including features of cellular senescence and increased susceptibility to apoptosis.

In summary, we demonstrate that sustained HLA-DM expression in RSV-infected CD1c^+^ DCs is a consequence of inhibited type I IFN signaling. We identify TBK1-dependent signaling as a regulator of HLA-DM expression during DC maturation and present evidence that variation in HLA-DM levels may influence downstream T cell phenotype at the DC priming stage. These findings establish a previously unrecognized link between innate antiviral signaling and antigen presentation machinery in human DCs, with potential implications for understanding RSV-associated immune dysfunction that may be further manipulated to modulate DC repertoire and improve patient and vaccine responses.

## Results

### HLA-DM is maintained in RSV infected CD1c^+^ DCs

**Figure 1:**
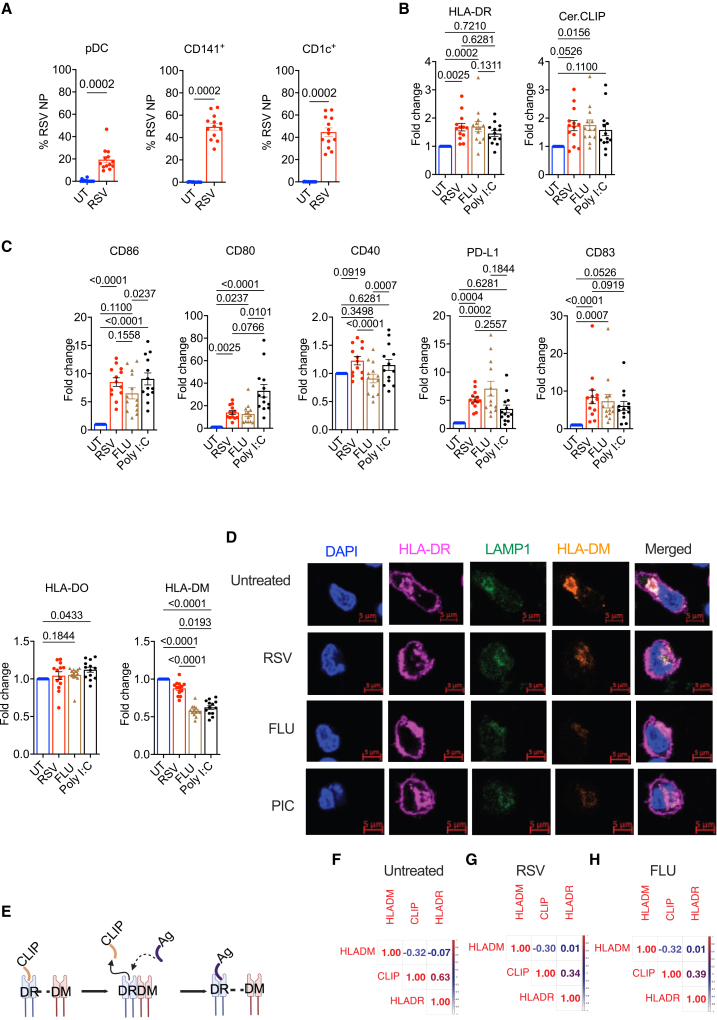
RSV maintains HLA-DM in CD1c^+^ DCs Blood DCs were treated with RSV, FLU, and PIC for T = 18 h, stained and analyzed using flow cytometry, median ±SEM. Fold-change plotted in all figures here forth represents the ratio of MFI in the treated condition relative to the untreated (medium) control for each donor, normalizing for inter-donor baseline variation Statistical comparisons not annotated are *p* > 0.9999. (A) Percentage RSV-NP^+^ from CD1c^+^, CD141^+^ and pDC. Wilcoxon matched pairs signed rank test used, *N* = 13. Data shown as median ± SEM (B and C) Fold change of HLA-DR, Cer.CLIP, CD86, CD80, CD40, PD-L1, CD83, HLA-DM, and HLA-DO of RSV, FLU and PIC infected CD1c^+^ DCs normalized to untreated cells. Data represent four independent experiments, *N* = 13. Also see Figure S1B. Data shown as median ± SEM (D) Confocal analysis on UT, RSV, FLU and PIC CD1c^+^ DCs stained for nucleus (Hoechst), HLA-DR, Lamp1, HLA-DM. Images were exported using Zenblue (Zeiss). Data represent two independent experiments. Scale bars represent 5 microns (μm), *N* = 3. Also see Figure S1C. Data shown as median ± SEM (E) Illustration of HLADR-HLADM interactions during antigen peptide exchange with endogenous peptide, CLIP. Created with BioRender.com. (F–H) Correlation plots of UT, RSV, and FLU treated CD1c^+^ DCs with markers examined in flow cytometry. Fishers Z transformation used for correlation calculations. See also Figure S1.

Blood DCs comprise myeloid DCs (mDCs) and plasmacytoid DCs (pDCs), characterized by distinct cell surface markers.^30^ mDCs are subdivided to CD1c^+^ or CD141^+^, the latter a minority subset^31^ (See Figures S1A and S1B). The effect of RSV infection on blood DC subsets was initially examined using flow cytometry using a high dimensional panel of DC markers. Our data showed that mDCs preferentially internalized more RSV compared to pDCs (Figure 1A). Further examination of the more frequent CD1c^+^ mDC population revealed similar activation phenotypes following stimulation with RSV, FLU, or poly I:C (PIC), characterized by increased surface MHCII HLA-DR, self-peptide CLIP, and activation markers CD86, CD80, CD40,PD-L1, and CD83 (Figures 1B and 1C, see Figure S1B). No changes were observed in HLA-DO expression across all conditions examined (Figure 1D).^17^ However, activation with FLU or PIC resulted in a downregulation of HLA-DM, in contrast to RSV-infected CD1c^+^ DCs where it was maintained (Figure 1D, see Figure S1C).

Next, we examined the requirement of live virus in mediating the changes in cell surface molecules. Heat killing RSV (RSVHK) did not affect its uptake into CD1c^+^ DCs when compared to live RSV conditions, despite completely losing its infection capacity in HEp-2 cells (see Figures S1D–S1F). RSVHK-infected CD1c^+^ DCs also demonstrated increased MHCII, CLIP, activation marker expression and retained HLA-DM expression (see Figures S1G–S1K).

### HLA-DM is maintained within MIIC and is functional during RSV infection

After translation, HLA-DM translocates from the endoplasmic reticulum to the late lysosomal organelle, MHCII like compartment (MIIC), where antigen presentation occurs.^11^ LAMP1 protein is expressed in the MIIC where autophagosomes containing digested peptides fuse for antigen loading.^22^^,^^23^^,^^32^^,^^33^ To assess whether the effects of RSV impacted this process, we examined MIIC associated HLA-DM expression following exposure to RSV, FLU, or PIC using confocal microscopy. Colocalization of LAMP1 and HLA-DM was observed in all conditions, indicating that HLA-DM was MIIC-bound (Figure 1E). Concordant with our flow cytometry dataset, HLA-DM intensity was highest in untreated cells which decreased significantly in FLU and PIC infected CD1c^+^ DCs. Similarly, RSV-infected DCs, retained their high HLA-DM intensity, suggesting increased antigen loading (Figure 1E).

RSV immunodominant epitopes are associated with HLA-DR,^34^ highlighting the importance of HLA-DM in facilitating removal of self-peptide CLIP and load antigen during antigen presentation (Figure 1F).^35^ Therefore, we performed correlation analysis on intracellular protein expression using the flow cytometry data. We determined a negative association between HLA-DM and CLIP in untreated, RSV and FLU infected CD1c^+^ DCs, illustrating that higher HLA-DM expression correlates with CLIP displacement, regardless of DC activation (Figures 1G–1I). A weaker positive correlation between CLIP and HLA-DR in RSV and FLU-infected CD1c^+^ DCs suggested increased peptide exchange following antigen driven activation (Figures 1G–1I).

### RSV infected CD1c^+^ DCs severely attenuate type I interferon responses

The regulation of MHCII-family gene expression is governed by regulatory factor X1 (RFX1) and class II transactivator (CIITA).^11^ Additionally, the opposing protein expression trends between surface HLA-DR and HLA-DM in FLU and PIC-infected, compared to RSV- infected CD1c^+^ DCs described above suggested additional mechanisms influencing HLA-DM expression. To investigate this, we examined changes in RNA transcriptional signatures over time in blood DCs post RSV and FLU infection using single cell RNA (scRNA) sequencing coupled with oligonucleotide antibodies (scAbSeq). In this second sample set, flow cytometry confirmed the HLA-DM signatures following activation described in Figure 1 (see Figure S2A). Using the Seurat analysis pipeline,^36^ we performed clustering and UMAP projection of all cells that were sequenced at 0 and 4 h, to establish changes in gene signatures at this early infection timepoint (see Figure S2B). CD1c^+^ DCs were identified from AbSeq (see Figure S2C) excluding RNA transcripts of major lineage markers corresponding to B cells (*MS4A1*), monocytes (*CD14*, *FCRGR3A*, *FCER1A*) and CD141^+^ DCs (*THBD)* (see Figures S2D–S2J). This analysis revealed that of the 26 clusters as visualized by UMAP (see Figure S2B), clusters 3, 5, 7, and 12 were CD1c^+^ DCs.

**Figure 2:**
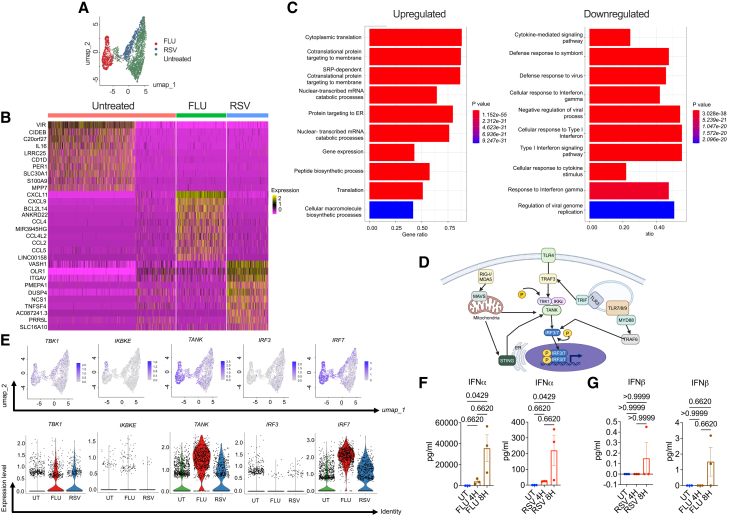
Type I interferon (IFN) pathway is severely attenuated in CD1c^+^ DC during RSV infection Blood DCs were infected with RSV or FLU from T = 0 h and 4 h before Abseq + scRNA sequencing. (A) UMAP representing scRNAseq transcripts from T = 0 h and 4 h of CD1c^+^ DCs according to treatment UT, RSV and FLU. Also see Figures S2K and S2L. (B) Heatmap of top 20 DEGs of UT, RSV and FLU CD1c^+^ DCs. (C) GOpathway analysis DEGs with 2-fold change cutoff of upregulated (blue) pathway and downregulated (red) pathways of RSV compared against FLU infected cells, normalized to untreated cells. (D) Illustration of a simplified canonical type I IFN pathway. (E) Feature heatmap output and violin plots of expression levels corresponding to RNA transcripts representing key regulators of the type I interferon pathway across all CD1c^+^ DCs. (F and G) Supernatant analysis of secreted IFNα and IFNβ of UT, RSV and FLU CD1c^+^ DCs used for scRNA analysis plotted as a timecourse from T = 0 (represented as untreated [UT]), 4 h and 8 h, *N* = 3. Data shown as median ± SEM See also Figures S2 and S3.

CD1c^+^ DCs were further characterized by clustering according to treatment conditions and their individual donor contributions to the resulting UMAP (Figure 2A, see Figures S2K and S2L). Differential gene expression (DEG) analysis of the top ten gene features of each condition indicated upregulation of genes associated with angiogenesis and T cell co-stimulatory markers e.g., *VASH1*,^37^^,^^38^ *TNFSF4*^39^^,^^40^ in RSV-infected cells. In contrast, genes associated with type I IFN response e.g., *CXCL11*, *CXCL9*, *CCL2*,^41^^,^^42^ or pro-inflammation were increased in FLU-infected DCs (Figure 2B).

To further investigate the heterogeneity observed within untreated CD1c^+^ DCs (Figure 2B), we performed focused subclustering at higher resolution, resolving four transcriptionally distinct subclusters (see Figures S3A and S3B). Cluster 0 expressed classical CD1c^+^ DC markers (CD1C, NDRG2, and PLCB2), whereas cluster 1 was defined by IFN -stimulated genes (CXCL11, CXCL10, IDO1, and RSAD2), consistent with an IFN-activated state. Cluster 2 was enriched for monocyte-associated transcripts (VCAN, FCN1, S100A8, and S100A9), suggesting the presence of a CD14^+^ inflammatory DC subset within the CD1c^+^ compartment, while cluster 3 expressed maturation-associated genes (VEGFA, DUSP4, TNFSF4, and IL7R).

To further resolve the two distinct clusters observed in untreated cells, we subsetted and reclustered only untreated CD1c^+^ DCs, identifying three subpopulations (see Figures S3C and S3D). Cluster 0 represented canonical cDC2 cells, enriched for MHC class II genes (HLA-DQA1, HLA-DQA2, and HLA-DOB) and CD1c^+^ DC markers. Cluster 1 displayed monocyte-lineage features (VCAN, FCN1, S100A8/A9, CSF1R), consistent with the previously described CD14^+^ DC3 subset.^43^^,^^44^ Cluster 2, the smallest population, expressed IFN -stimulated genes (MX1, CXCL10, IFIT1/2/3, HERC5), indicating a basally IFN-primed state. Cluster separation was not driven by mitochondrial RNA content (see Figures S3C and S3D), and all donors contributed to each cluster, supporting biological rather than technical heterogeneity.

Importantly, this CD14^+^ inflammatory DC subset represented a low-frequency population within the CD1c^+^ compartment, and is therefore unlikely to substantially influence the overall transcriptional signatures observed. Furthermore, all functional analyses were performed on a stringently gated live CD3^−^CD19^−^CD56^−^CD14^−^CD16^−^CD11c^+^CD1c^+^ population by flow cytometry, excluding CD14^+^ and CD16^+^ cells and ensuring that the primary findings reflect bona fide CD1c^+^ DC biology. As such, while scRNA-seq reveals transcriptional heterogeneity within the CD1c^+^ DC compartment, this does not confound the functional conclusions derived from the purified CD1c^+^ DC population.

RNA levels of HLA-DM remained unchanged between RSV and FLU-infected DCs, suggesting the changes observed earlier were at the protein level (see Figures S3E and S3F). We then conducted gene pathway analysis of DEGs using the call to DEenrichRPlot^45^^,^^46^^,^^47^^,^^48^ in Seurat between RSV and FLU infected cells. In agreement with the analysis above, the type I IFN (IFN) pathway was higher in FLU infected DCs compared to those exposed to RSV (Figure 2C). Type I IFN signaling requires phosphorylation of the complex formed between TRAF family member-associated NF-κB activator (TANK), TANK-binding kinase 1 (TBK1) and the inhibitor of nuclear factor kappa-B kinase subunit epsilon (IKKε) for transcription of type I IFNα/β through IFN regulatory factor (IRF) 3 and IRF7 (Figure 2D).^49^^,^^50^^,^^51^ Selected UMAP visualization of these genes showed that while *TBK1*, *IKK*ε, and *IRF3* remained similar across untreated, RSV and Flu stimulations, RSV clusters expressed less *TANK*, *IRF7* RNA, showing similar expression to untreated samples (Figure 2E).

Secreted IFN α and β and levels in culture supernatants were measured by multiplex ELISA, with background-subtracted fluorescence converted to concentrations using standard curves (Figures S3G and S3H; see Table S1A); out-of-range values were set to zero. IFNα was undetectable in untreated cells (T = 0 h) and remained largely below the detection limit in RSV-infected CD1c^+^ DCs at 4 and 8 h, whereas Flu-infected DCs showed detectable IFNα at 4 h (Figure 2F). Similarly, IFNβ was undetectable in RSV-infected cultures at both timepoints but detectable in Flu-infected DCs, particularly at 8 h (Figure 2G). Although low-level IFNβ production below the detection limit cannot be excluded, such levels are unlikely to drive meaningful downstream signaling.

Our findings in these *ex vivo* primary CD1c^+^ DCs is supported by previous studies demonstrating a lack of a type I IFN response following RSV infection in epithelial A549 cell lines, macrophages, and monocyte derived DCs.^24^^,^^25^^,^^28^^,^^52^ The absence of type I IFN secretion despite preserved expression of pathway components suggests that RSV disrupts a specific step in the signaling cascade rather than broadly suppressing pathway gene expression. Type I IFN (IFN-I) production downstream of RIG-I/MDA5-TBK1-IRF3/7 signaling is known to be antagonized by RSV NS1/NS2 proteins. Hence, reduced IFN-I signaling during RSV infection may therefore prevent the HLA-DM downregulation that normal accompanies DC maturation.

### RSV blocks IRF3 nuclear translocation to inhibit type I IFN production and maintain HLA-DM expression

**Figure 3:**
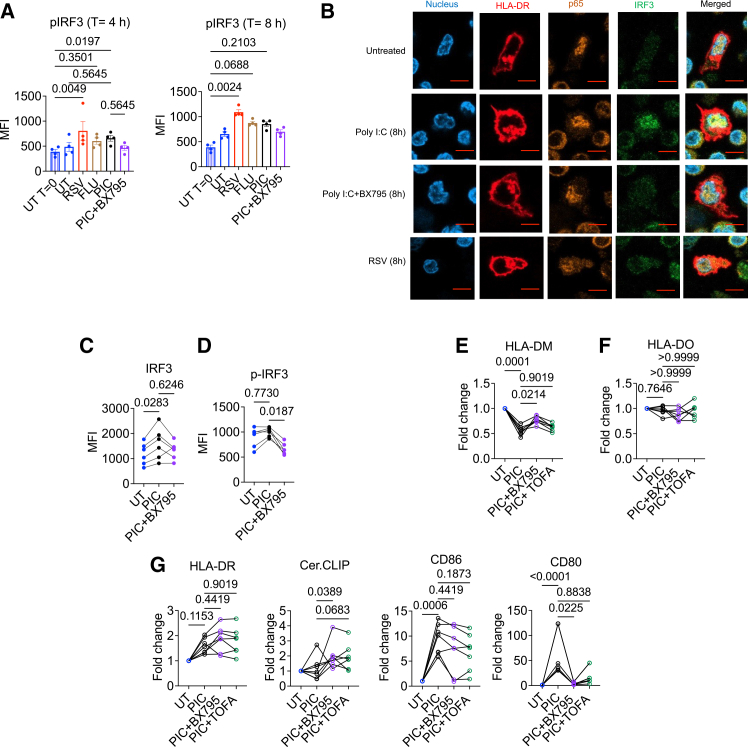
TBK1 associated HLA-DM regulation is independent of DC activation and JAK/STAT signaling CD1c^+^ DCs were left UT or stimulated with PIC in the presence or absence of the TBK1 inhibitor BX795 or the JAK inhibitor TOFA. Data represent independent experiments across multiple donors, shown as median ±SEM (A) MFI of phosphorylated IRF3 (pIRF3) in CD1c^+^ DCs following stimulation with RSV, FLU, or PIC with or without BX795 at the indicated time points T = 0, 4, and 8 h. Also see Figures S4A and S4B. (B) Confocal microscopy of CD1c^+^ DCs following stimulation with PIC, PIC+BX795, or RSV for 8 h. Cells were stained for nucleus (Hoechst), HLA-DR, NF-κB p65, and IRF3 to assess subcellular localization. Scale bars represent 5 microns (μm). (C and D) MFI quantification of IRF3 and pIRF3 expression in CD1c^+^ DCs stimulated with PIC in the presence or absence of BX795 at 2 h. Also see Figures S4C and S4D. (E–G) Fold change of HLA-DM and HLA-DO, HLA-DR, CLIP, CD86, and CD80 of CD1c^+^ DCs stimulated with PIC, with or without BX795, *N* = 7. Also see Figures S4F, S4G, and S5A. See also Figure S4.

To identify the step at which RSV disrupts type I IFN signaling, we examined the phosphorylation status of key pathway components: pTBK1, pIRF3, and pIRF7. Interestingly, we show that pTBK1 was slightly reduced, while pIRF3 and pIRF7 phosphorylation remained intact in RSV infected CD1c^+^ DCs (Figure 3A, see Figures S4A and S4B). This demonstrates that RSV did not prevent phosphorylation of key transcription factors.

Since phosphorylation of IRF3 is necessary but not sufficient for type I IFN transcription, we next examined IRF3 subcellular localization using confocal microscopy. Strikingly, while NF-κB signaling remained intact in RSV-infected CD1c+ DCs, IRF3 nuclear localization was completely abrogated (Figure 3B). This represents a critical finding, despite normal phosphorylation, pIRF3 fails to translocate to the nucleus in RSV-infected cells, thereby preventing type I IFN gene transcription. This observation is consistent with the known mechanism of RSV NS1, which binds IRF3 and its transcriptional coactivator CBP, preventing their association and inhibiting IRF3-dependent transcription without affecting IRF3 phosphorylation.^25^^,^^52^ These data suggest that RSV selectively blocks IRF3 function while preserving other aspects of innate signaling, including NF-κB-dependent inflammatory responses.

Given that RSV infection specifically disrupts IRF3 nuclear localization downstream of TBK1 while maintaining HLA-DM expression, we hypothesized that TBK1 signaling regulates HLA-DM levels. To test this, we inhibited TBK1 using BX795 in PIC-stimulated CD1c^+^ DCs. In parallel, we used the JAK/STAT inhibitor tofacitinib (TOFA) to block the activity of secreted IFNα/β. As expected, BX795 reduced pIRF3 levels during PIC stimulationc (Cohen’s d = 3.24), confirming inhibition of TBK1 signaling (Figures 3C and 3D, see Figures S4C–S4D).^53^ Furthermore, BX795 treatment prevented the downregulation of HLA-DM (Cohen’s d = 2.52), while HLA-DO expression was statistically insignificant but by Cohen’s d calculations modestly affected (*p* > 0.9999, Cohen’s d = 0.738) (Figure 3E). A second TBK1 inhibitor, MRT67307, produced a similar effect (Cohen’s d = 5.44), confirming the specificity of TBK1 signaling in regulating HLA-DM expression (see Figure S4E). BX795 and TOFA inhibit upstream type I IFN signaling and secreted IFN activity, respectively, both of which contribute to DC activation.^54^^,^^55^^,^^56^ In PIC-stimulated CD1c^+^ DCs, BX795 and TOFA increased CerCLIP levels (Cohen’s d = 0.973 and 0.952 respectively) while reduced PD-L1 (Cohen’s d = 2.03 and 2.35 respectively) and CD80 expression (Cohen’s d = 2.41 and 1.29 respectively) without affecting HLA-DR (Cohen’s d = 0.705 and 0.460 respectively) or CD86 (Cohen’s d = 0.561 and 0.794 respectively) (Figure 3G, see Figures S4F, S4G, and S5A), indicating preservation of core antigen presentation machinery. Although a directional reduction in CD40 expression was observed with BX795, this did not reach statistical significance (see Figure S4F, *p* = 0.1153 Cohen’s d = 0.905; see Figure S5A, *p* > 0.999, Cohen’s d = 0.843) and was modest relative to HLA-DM. These data argue against a global defect in DC activation and instead support a selective effect on antigen processing pathways, consistent with HLA-DM-mediated modulation of T cell responses. Similar trends with MRT67307 support a TBK1-associated effect. Moreover, BX795, TOFA, and MRT67307 had no direct effect on any CD1c^+^ DC marker expression examined above (see Figure S5B). Consistent with a role for TBK1 in regulating HLA-DM, addition of the TBK1 inhibitor BX795 to influenza-infected CD1c^+^ DCs produced only a modest increase in HLA-DM expression (*N* = 5 donors; see Figure S4H). Influenza activates TBK1 but also engages multiple innate pathways, including TLR7/8 and RIG-I, likely limiting the effect of TBK1 inhibition. In contrast, PIC signals primarily through the TLR3–TRIF-TBK1 axis, consistent with the stronger HLA-DM rescue observed under PIC+BX795 conditions. Together, these findings indicate that HLA-DM downregulation requires activation of both NF-κB and TBK1 signaling, independent of DC activation and JAK/STAT signaling, revealing a link between innate antiviral signaling and antigen presentation machinery. HLA-DM functions as a peptide editor within the MHC-II antigen-processing pathway. During normal DC maturation, HLA-DM expression decreases, allowing stable peptide-MHC-II complexes to accumulate. Sustained HLA-DM expression, as observed during RSV infection, may maintain ongoing peptide editing and alter peptide loading.

By blocking IRF3 nuclear translocation, RSV may simultaneously suppress type I IFN production while maintaining elevated HLA-DM expression, potentially broadening the DC peptide repertoire and altering downstream T cell responses.^15^^,^^16^^,^^17^

### HLA-DM^hi^ DCs expand less terminally differentiated CD73^−^ CD39^+^ virus-specific T cells with dampened effector responses

**Figure 4:**
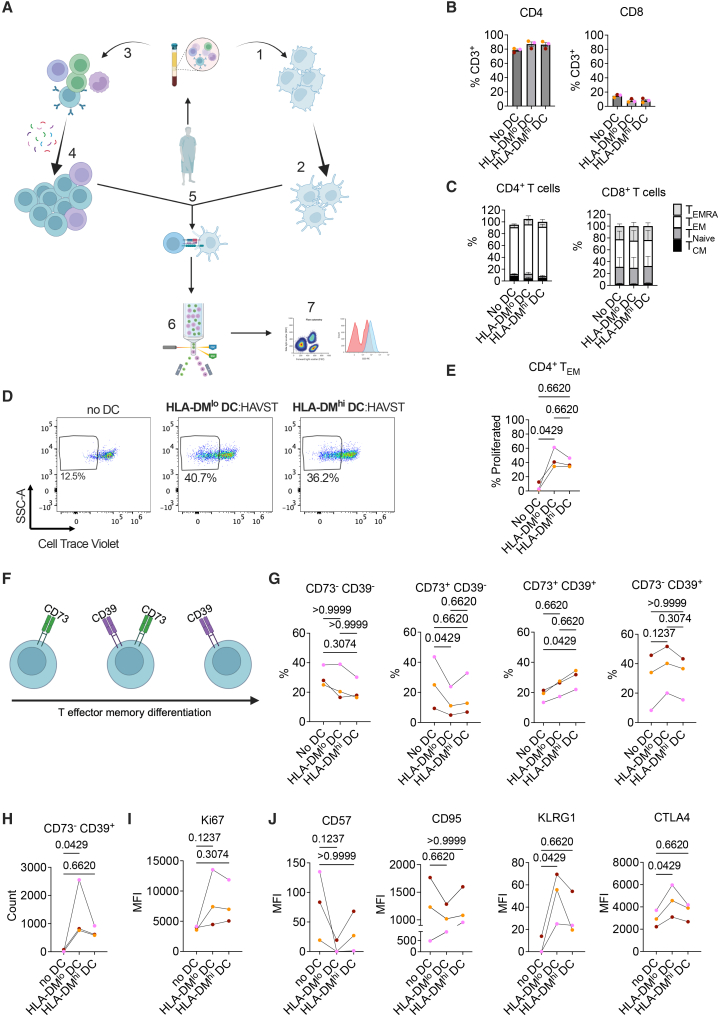
HLA-DM^hi^ DCs expand HAVST_em_ associated with altered function Functional assays of CD1c^+^ DC are readout against full length Influenza A H1N1 (A/California/07/2009) HA. Two CD1c^+^ DC states were generated by treatment of PIC+BX795 (HLA-DMhi) or PIC (HLA-DMlo) alone and evaluated against a coculture with autologous HA virus specific T (HAVST) cells 5 days post coculture, *N* = 3, data shown as median ± SEM. (A) Illustration of experimental setup. CD1c^+^ DCs were positively isolated and primed with HA peptide (steps 1–2). Autologous HAVSTs were generated from PBMCs (steps 3–4). The resulting HA primed CD1c^+^ DCs and HAVSTs were cocultured (step 5) and flow cytometry analysis performed 5 days after (step 6 and 7). Also see Figures S5D and S5E. Created with BioRender.com. (B) Percentages of total CD4^+^ and CD8^+^ HAVST cells against CD3^+^ T cells. (C) Phenotypical analysis of CD4^+^ and CD8^+^ VST cells stratified into effector memory HAVST_em_ (T_em_), exhausted HAVST_emra_ (T_emra_), Naive HAVST (T_naïve_) and central memory HAVST cells (T_cm_). (D) Side scatter (SSC-A) of cell trace violet (CTV) stained CD4^+^ T cells of a representative donor (denoted by brownish datapoints in all graphs). (E) Line plot of percentage proliferated CD4^+^ T cells from CTV gating. (F) Illustration of T cell effector memory differentiation. Created with BioRender.com. (G) Percentage of CD73 and CD39 expression of CD4^+^ T_em_ cells expressed as CD73^−^CD39^−^, CD73^+^ CD39^−^, CD73^+^ CD39^+^, and CD73^−^ CD39^+^ respectively. (H–J) Cell count and MFI of Ki67, CD57, CD95, KLRG1 and CTLA4 of CD73^+^ CD39^+^ CD4^+^ T_em_ cells. See also Figure S5.

To assess the effect of HLA-DM expression on DC-T cell priming, we generated HLA-DM^hi/lo^ CD1c^+^ DCs using a treatment of PIC and BX795 (HLA-DMhi) or PIC alone (HLA-DMlo) (Figure 4A, step 1–2). Antigen presentation capacity was evaluated by co-culture with autologous antigen-primed T cells using full-length Influenza A H1N1 hemagglutinin (HA) protein to bias toward MHC-II presentation^57^^,^^58^^,^^59^ (Figure 4A). Using this HA protein, a pool of autologous HAVST (HA-primed virus-specific T cells) was generated from three haplotype-matched donors (see Figure S5C) and confirmed antigen specificity via secreted IFNγ (Figure 4A step 3–4, see Figure S5D).

Autologous HAVSTs and HA-primed HLA-DM^hi/lo^ CD1c^+^ DCs were co-cultured (Figure 4A step 5), with HAVST proliferation and phenotype evaluated using flow cytometry with specific T cell naive and memory cell surface markers (Figure 4A step 6–7, see Figure S5E). As expected, HAVSTs across all conditions were majority CD4^+^ T effector memory (T_em_:CD3+CD4^+^CD45RA^−^CCR7^+^) phenotype (Figures 4B and 4C). Notably, HLA-DM^hi^ DCs did not significantly enhance CD4^+^ HAVST_em_ proliferation compared to HLA-DM^lo^ DCs (Figures 4D and 4E).

From this point onward, analyses were restricted to proliferating CD4^+^ HAVST_em_ cells (HA-primed CD3^+^CD4^+^CD45RA^−^CCR7^-^) identified by CTV dilution, to specifically assess proliferation-dependent phenotypic changes during co-culture. Canonical VST differentiation is characterized by CD73 downregulation and CD39 upregulation (CD73^−^ CD39^+^), leading to terminally differentiated effector cells capable of cytokine secretion and effector response (Figure 4F).^60^^,^^61^^,^^62^

HLA-DM^hi^, but not HLA-DM^lo^ DC cultures showed lower percentages of CD73^−^CD39^+^ cells (Cohen’s d = 0.362) within the CD4^+^ HAVST_em_ subset. HLA-DM^hi^ induced CD73^−^CD39^+^ cells also exhibited decreased proliferation (Cohen’s d = 0.643), higher CD57^63^^,^^64^ (Cohen’s d = 0.753) and CD95 (Fas) (Cohen’s d = 1.09) expression (Figures 4G–4J). A minor CD73^−^CD39^−^ HAVSTem population was also observed, consistent with previous reports of uncertain functional significance^61^^,^^62^ (Figure 4G). While these trends suggest impaired differentiation and increased susceptibility to apoptosis, they are based on three donors and will require further validation in larger cohorts.

Together, these findings suggest that HLA-DM^hi^ DCs may impair HAVSTem differentiation and promote features associated with reduced effector function, cellular senescence, and Fas-associated apoptosis.^65^^,^^66^ These observations are consistent with a model in which sustained HLA-DM expression alters antigen presentation and influences CD4^+^ T cell priming, though confirmation in larger cohorts will be important.

In summary, our study identifies a previously unrecognized role for type I IFN signaling in regulating HLA-DM expression in CD1c^+^ DCs, providing a link between TBK1-dependent innate signaling and antigen presentation machinery. Co-culture experiments suggest that HLA-DM^hi^ DCs are associated with altered T cell differentiation phenotypes, consistent with a model in which sustained HLA-DM expression may influence peptide repertoire resulting in downstream T cell responses during RSV infection.

## Discussion

Understanding poor adaptive immune responses in RSV infection is crucial for therapeutic targeting. We addressed this by studying RSV’s impact on CD1c^+^ DC function. Our findings reveal maintained HLA-DM expression in RSV infected DCs, resulting from inhibited type I IFN pathway.

Through pharmacological modification of HLA-DM expression, we observed that HLA-DM^hi^ DC is associated with altered CD4^+^ T_em_ cell differentiation, including features of increased cellular senescence and apoptosis associated receptors. This observation is consistent with the potential effect HLA-DM maintenance may have on CD4 T_em_ cells. Given HLA-DM’s role in DC peptide loading, we postulate that RSV-infected CD1c^+^ DCs, like HLA-DM^hi^ CD1c^+^ DCs, display a broadened peptide repertoire which profoundly impacts downstream T cell immunogenicity.^12^^,^^13^

HLA-DM is a non-classical MHC class II molecule that functions as a peptide editor within the endosomal antigen-processing pathway. During normal DC maturation, reduced HLA-DM expression allows high-stability peptide-MHC-II complexes to accumulate and efficiently stimulate antigen-specific CD4^+^ T cells. In contrast, sustained (or high) HLA-DM expression, as observed during RSV infection, may maintain ongoing peptide editing, reducing the relative density of individual peptide-MHC-II complexes and resulting in a broader but less focused peptide-MHC-II repertoire. This altered peptide display is expected to weaken peptide-specific TCR signaling. Consistent with this model, our CerCLIP data (Figure S4G), which measure the expression of HLA-DR-associated CLIP complexes, demonstrate altered peptide loading following modulation of HLA-DM expression. In line with this, HLA-DM-high DCs induced CD4^+^ T cells with reduced effector differentiation, supporting a mechanistic link between HLA-DM-dependent antigen processing and impaired T cell responses.

RSV-infected CD1c^+^ DCs displayed an activated phenotype,^7^^,^^67^^,^^68^ yet maintained HLA-DM expression that is inconsistent with canonical DC activation.^17^ HLA-DO, the natural inhibitor of HLA-DM function,^18^ remained unchanged across all conditions, further suggesting that RSV-infected DC peptide selection is not driven by enhanced or diminished HLA-DO function. Consistent with this, high HLA-DM expression has been associated with increased nonspecific DC peptide repertoire.^19^^,^^20^^,^^69^ This suggests that peptide variety and subsequent loading on HLA-DR can reduce epitope density, lessen TCR-peptide interactions and cumulatively shorten TCR-pMHCII binding kinetics, leading to reduced T cell responses.^12^^,^^13^^,^^68^ Therefore, although peptide repertoire on RSV infected DCs was not examined in our study, it is conceivable that higher HLA-DM RSV infected DCs would expand T cells with weaker affinity TCRs, delaying anti- RSV T cell effector responses.^70^^,^^71^^,^^72^^,^^73^

Our scRNA sequencing and supernatant analysis demonstrated that RSV infected CD1c^+^ DCs inhibited type I IFN signaling, a seemingly common consequence of RSV infection in other cell types.^74^^,^^75^ We further showed RSV did not hinder phosphorylated TBK1/IRF3/IRF7 or NF-κB signaling but inhibited IRF3 nuclear localization in CD1c^+^ DCs, revealing that HLA-DM downregulation requires both NF-κB and type I IFN signaling to occur. To confirm the dependence of type I IFN signaling on HLA-DM regulation, we employed BX795, a selective inhibitor of TBK1 signaling. BX795 prevented HLA-DM downregulation induced by PIC or MDA5/RIG-I, both of which are known to be involved in RSV recognition.^75^ This finding further supports the critical role of the type I IFN pathway in modulating HLA-DM expression during RSV infection.

While genetic approaches such as HLA-DM knockdown or overexpression would provide definitive causal evidence and represent an important future direction, concordant effects from two TBK1 inhibitors, together with changes in CerCLIP, support a TBK1-HLA-DM regulatory axis linking TBK1 signaling to antigen presentation and suggesting a mechanism by which RSV-mediated disruption of TBK1-IRF3 signaling may alter T cell priming.

Previous research implicates RSV NS1 in directly inhibiting pIRF3 nuclear translocation to affect type I IFN transcription.^24^^,^^26^^,^^27^^,^^52^ Therefore, we propose that RSV inhibits type I IFN signaling to achieve a dual outcome of suppressing IFN secretion and limiting antigen presentation through high HLA-DM expression in DCs.

Interestingly, BX795/MRT67307 has been shown to inhibit autophagy through ULK1^76^ and HLA-DM undergoes lysosomal degradation through MARCH E3 ligase ubiquitination,^77^ implying autophagy driven effects of these TBK1 inhibitors on HLA-DM expression. Hence, to establish the role of autophagy in HLA-DM regulation, we further incubated rapamycin^78^ and bafilomycin A1^79^ with PIC in CD1c^+^ DCs. Preliminary findings showed that HLA-DM expression was unhindered in autophagy inducing or inhibiting conditions, suggesting that TBK1 signaling specifically regulates HLA-DM possibly through a separate mechanism (see Figure S5H). Combined, we postulate that HLA-DM downregulation is likely a canonical effect of DC activation, driven by type I IFN signaling and uniquely inhibited in RSV infection, establishing a previously unknown link between innate and adaptive immune responses.

During RSV infection, poor T cell responses are characterized by low proliferation and lymphopenia,^4^^,^^80^ yet we showed that while HLA-DM^hi^ DCs displayed a reduced proliferation trend, they exhibited a phenotype akin to hindered terminal differentiation (CD73^−^ CD39^+^) of CD4 HAVST_em_^60^^,^^61^^,^^62^^,^^81^ This suggests that HLA-DM induced wider DC peptide repertoire has a larger influence on HAVST effector function and phenotype more than proliferation. Within the HLA-DM^hi^ DC primed terminally differentiated CD4^+^ HAVST_em_ subset, cells exhibited a trend of diminished proliferative capacity and heightened predisposition for CD95 (Fas) associated apoptosis, which is consistent with higher Fas and TRAIL mediated T cell apoptosis in patients suffering from acute RSV infection.^80^ Further analysis illustrated that these CD4^+^ HAVST_em_ reduced checkpoint inhibitor KLRG1 and CTLA4 expression, suggesting a differential effect that widened DC peptide repertoire has on T cell responses.^82^ Attenuated T cell responses, despite manifesting an activated DC phenotype, have been documented in Measles infection.^83^^,^^84^ Collectively, our data illustrates a potentially clinical relevant T cell subset as a potential consequence of HLA-DM^hi^ expression on DC peptide repertoire. This reinforces our assertion that in RSV infection, widened DC peptide repertoire may contribute more to overall impaired adaptive responses, driving poor anti-viral immune response.

In summary, our study reveals that a sustained HLA-DM expression is a distinctive phenotype of RSV infected DCs, arising from inhibition of the type I IFN signaling pathway. We identify TBK1-dependent signaling as a regulator of HLA-DM expression during DC maturation, supported by dual inhibitor concordance, cross-pathway conservation, and exclusion of alternative mechanisms. CerCLIP data confirm that modulation of HLA-DM levels alters peptide loading at the DC surface. Co-culture experiments suggest that HLA-DM levels may influence T cell differentiation phenotype, consistent with known roles of HLA-DM in shaping peptide repertoire and TCR-pMHCII interactions. The manipulation of HLA-DM levels through the TBK1-IFN axis may have broader implications for disease scenarios with aberrant T cell responses. Our findings establish a previously unrecognized link between innate antiviral signaling and antigen presentation machinery in human DCs, contributing to our understanding of RSV pathogenesis and suggesting directions for future investigation of DC-targeted therapeutic strategies.

### Limitations of the study

This study has several limitations. Our experiments use *ex vivo* blood-derived CD1c^+^ DCs, which may not fully capture the airway mucosal environment where RSV infection occurs. Donor variability, partly influenced by HLA-DRB1 polymorphism, also contributes to heterogeneity in immune responses. Notably, all three donors shared HLA-DRB1∗09:01:02, which was intended to serve as a common HLA-DR restricting element for HA peptide presentation and improved comparability across co-culture conditions, consistent with the HLA-DR restriction of immunodominant CD4^+^ T cell epitopes. The presence of distinct second HLA-DRB1 alleles in each donor nevertheless preserves inter-individual variation in peptide binding properties. As HLA-DR polymorphism influences peptide binding stability and susceptibility to HLA-DM-mediated editing, more stable peptide-MHC-II complexes tend to resist peptide exchange, whereas less stable complexes remain permissive to continued editing, thereby shaping peptide repertoire diversity and influencing CD4^+^ T cell priming. Finally, validation in airway-resident DC subsets and *in vivo* models will be important to determine the broader relevance of this TBK1–HLA-DM regulatory mechanism in RSV immunity.

## Resource availability

### Lead contact

Requests for further information and resources should be directed to and will be fulfilled by the lead contact, John Edward Connolly (jeconnolly@a-star.edu.sg).

### Materials availability

This study did not generate new unique reagents.

### Data and code availability

• scRNAseq data and the processed count matrices have been deposited at GEO as GSE334417 and are publicly available at https://www.ncbi.nlm.nih.gov/geo/query/acc.cgi?acc=GSE334417. All code used to analyze the scRNAseq data has been deposited at https://doi.org/10.5281/zenodo.20546842. For additional convenience, a copy of the code and processed scRNAseq data used in the manuscript has also been made available at https://github.com/EnJun-Yang/code_for_publications/tree/main/iscience_2026.
• Any additional information required to reanalyze the data reported in this study is available from the lead contact upon request.

## Acknowledgments

We sincerely thank Anna Marie Fairhurst for their input and assistance. This study was supported by the core funding from Institute of Molecular and Cell Biology (10.13039/501100007674IMCB), A∗STAR Singapore.

## Author contributions

W.O. and J.E.C. designed and directed the study. W.O., E.Y., N., N.T., and B.A. set up and performed experiments. E.Y. performed computational analysis. W.O., E.Y., N., N.T., B.A., R.A.H., and J.E.C. analyzed and interpreted the data. W.O., R.A.H., E.Y., and J.E.C. wrote the manuscript.

## Declaration of interests

The authors declare no competing interests.

## STAR★Methods

### Key resources table

**Table:** 

REAGENT or RESOURCE	SOURCE	IDENTIFIER
**Antibodies**		

CD3 antibody (clone UCH1), PECF594	BD Biosciences	Cat#562280; RRID: AB_11153674
CD19 antibody (clone HIB19), PECF594	BD Biosciences	Cat#562294; RRID: AB_11154408
CD14 antibody (2255936, PECy5.5)	Life Technologies	Cat#MHCD1418; RRID: AB_10371748
CD56 antibody (clone NCAM16.2), PECF594	BD Biosciences	Cat#564849; RRID: AB_2738983
CD16 antibody (clone 3G8), BUV496	BD Biosciences	Cat#564653; RRID: AB_2744294
CD11c antibody (clone B-Ly6), BUV661	BD Biosciences	Cat#612967; RRID: AB_2870241
CD123 antibody (clone 9F5), PECy5	BD Biosciences	Cat#551065; RRID: AB_394029
BDCA4 antibody (clone Neuropilin U21-1283, BV605)	BD Biosciences	Cat#743130; RRID: AB_2741297
CD1c antibody (clone F10/21A3), BUV805	BD Biosciences	Cat#748722; RRID: AB_2873126
CD141 antibody (clone 1A4, BV711)	BD Biosciences	Cat#563155; RRID: AB_2738033
HLA-DR antibody (clone G46-6), V500	BD Biosciences	Cat#561224; RRID: AB_10563765
CD80 antibody (clone l307.4), BB790	BD Biosciences	RRID: AB_3752222
CD83 antibody (clone HB15e), BUV786	BD Biosciences	Cat#565336; RRID: AB_2739191
CD86 antibody (clone FUN-1), BUV737	BD Biosciences	Cat#612784; RRID: AB_2814790
ILT3 antibody (clone ZM4.1), PECy7	BioLegend	Cat#333012; RRID: AB_2564067
PD-L1 antibody (clone MIH1), BV421	BD Biosciences	Cat#563738; RRID: AB_2738396
CLIP antibody (clone Cer.CLIP), BUV395	BD Biosciences	Cat#742601; RRID: AB_2740901
CD40 antibody (clone 5C3, Alexa Fluor 700)	BD Biosciences	Cat#561208; RRID: AB_10611570
HLA-DOβ antibody (clone DOB.L1), Alexa Fluor 546	Santa Cruz Biotechnology	Cat#sc-69739; RRID: AB_2012531
HLA-DM antibody (clone REA406, APC)	Miltenyi Biotec	Cat#130-122-957; RRID: AB_2801985
RSV antibody (clone I25J, FITC)	Invitrogen	Cat#MA1-7289; RRID: AB_1018350
Influenza antibody (clone D67J, FITC)	Abcam	Cat#ab210526; RRID: AB_3752219
MARCH8 antibody (polyclonal, PECy5)	Thermo Fisher Scientific	Cat#PA5-20632; RRID: AB_11157657
MARCH1 antibody (polyclonal, PE/Texas Red)	Thermo Fisher Scientific	Cat#PA5-144822; RRID: AB_3092325
MARCH9 antibody (clone 2B5, PECy7)	Thermo Fisher Scientific	Cat#H00092979-M01; RRID: AB_2140303
LAMP1 antibody (clone H4A3), Alexa Fluor 488	Abcam	Cat#ab187591; RRID: AB_2884958
IRF3 antibody (clone D9J5Q, AF488)	Cell Signaling Technology	Cat#67651S; RRID: AB_3752220
p65 antibody (clone D14E12, unconjugated)	Cell Signaling Technology	Cat#8242S; RRID: AB_10859369
Goat anti-Rabbit IgG H&L secondary antibody (Alexa Fluor 555)	Abcam	Cat#ab150078; RRID: AB_2722519
*p*-IRF3 (Ser396) antibody (clone D601M), PE	Cell Signaling Technology	Cat#83611S; RRID: AB_2800022
Lin1 antibody cocktail, FITC	BD Biosciences	Cat#340546; RRID: AB_400053
CD4 antibody (clone SK7, BV480)	BD Biosciences	Cat#566165; RRID: AB_2739562
CD8 antibody (clone SK1, BUV805)	BD Biosciences	Cat#612889; RRID: AB_2833078
CD45RA antibody (clone HI100)	BioLegend	Cat#304138; RRID: AB_2563815
CCR7 antibody (clone 3D12), PECy7	BD Biosciences	Cat#557648; RRID: AB_396765
CD25 antibody (clone 2A3), BUV395	BD Biosciences	Cat#564034; RRID: AB_2738556
PD-1 antibody (clone EH12.1), BUV737	BD Biosciences	Cat#612791; RRID: AB_2870118
CD69 antibody (clone FN50), BUV563	BD Biosciences	Cat#748764; RRID: AB_2873167
KLRG1 antibody (clone Z7-205.rMab), PECF594	BD Biosciences	Cat#568658; RRID: AB_3684443
CD95 antibody (clone DX2, Alexa Fluor 700)	BioLegend	RRID: AB_3752221
CD39 antibody (clone Tu66), BV605	BD Biosciences	Cat#742522; RRID: AB_2740840
CD73 antibody (clone AD2), BV786	BD Biosciences	Cat#742635; RRID: AB_2740928
CD57 antibody (clone NK-1), BUV615	BD Biosciences	RRID: AB_3752224
FOXP3 antibody (clone 259D/C7), PE	BD Biosciences	Cat#560046; RRID: AB_1645508
Ki67 antibody (clone Ki67, Alexa Fluor 488)	BioLegend	Cat#350508; RRID: AB_10933085
CTLA4 antibody (clone BNI3), PECy5	BD Biosciences	Cat#555854; RRID: AB_396177
MHC I (HLA-A, B, C) blocking antibody (clone W6/32)	BioLegend	Cat#311412; RRID: AB_493132

**Bacterial and virus strains**		

Respiratory Syncytial Virus (RSV)	ATCC	Cat#VR-1540P
Influenza A/PR/8/34	Charles River	Cat#10100374

**Biological samples**		

Human PBMCs from healthy donors	This study	IRB Ref#2019-095

**Chemicals, peptides, and recombinant proteins**		

Poly I:C	Miltenyi Biotec	Cat#130-112-563
Lipopolysaccharide (LPS)	Invitrogen/Life Technologies	Cat#00-4976-93
Hoechst trihydrochloride trihydrate	Thermo Fisher Scientific	Cat#33342
BX795	Axon Medchem	Cat#1390
MRT67307	MedChemExpress	Cat#HY-13018
Tofacitinib citrate	Tocris	Cat#4556
R848	InvivoGen	Cat#tlrl-r848-5
GM-CSF	Miltenyi Biotec	Cat#130-093-868
IL-4	Miltenyi Biotec	Cat#130-093-924
Flt3-L	Miltenyi Biotec	Cat#130-096-480
IL-1β	Miltenyi Biotec	Cat#130-093-898
IL-2	Miltenyi Biotec	Cat#130-097-744
IL-7	Miltenyi Biotec	Cat#130-095-362
IL-15	Miltenyi Biotec	Cat#130-095-766
Influenza A/04/09/California peptide mix	JPT Technologies	Cat#PM-INFA-HACal
Full-length HA peptide	Sino Biological	Cat#11085-V08B-100

**Critical commercial assays**		

CD3 MicroBeads	Miltenyi Biotec	Cat#130-097-043
CD19 MicroBeads	Miltenyi Biotec	Cat#130-119-475
CD14 MicroBeads	Miltenyi Biotec	Cat#130-050-201
CD56 MicroBeads	Miltenyi Biotec	Cat#130-097-042
Granulocyte Isolation Beads	Miltenyi Biotec	Cat#130-126-448
Human Blood Dendritic Cell Isolation Kit	Invitrogen	Cat#11308D
Near IR live/dead viability stain	Invitrogen	Cat#L34976
FOXP3/Transcription Factor Staining Buffer Set	Invitrogen	Cat#00-5523-00
BD Phosfix Buffer 1	BD Biosciences	Cat#557870
BD Phosflow Perm/Wash Buffer 1	BD Biosciences	Cat#557885
Milliplex 9-plex Human Interferon Panel	Merck Millipore	Cat#HIFN-130K-09

**Deposited data**		

scRNA-seq raw data and code	This paper	GEO: GSE334417; Zenodo: https://doi.org/10.5281/zenodo.20546842; https://www.ncbi.nlm.nih.gov/geo/query/acc.cgi?acc=GSE334417

**Software and algorithms**		

FlowJo v6.0	BD Biosciences	https://www.flowjo.com/
GraphPad Prism v9.5.1	GraphPad	https://www.graphpad.com/
R v4.2.2	R Foundation	https://www.r-project.org/
Seurat v4.3.0	Satija Lab	https://satijalab.org/seurat/
ZEN Blue	ZEISS	https://www.zeiss.com/

### Experimental model and study participant details

#### Peripheral blood mononuclear cell (PBMC) and dendritic cell (DC) isolation

Blood was taken from healthy donors in Singapore with informed consent (Institutional Review Board, Reference no.2019-095). Participant demographic data including sex, age, and ethnicity were not systematically collected for all samples, as de identified donor materials were obtained through institutional repositories under approved IRB above. Gender identity and socioeconomic status information were unavailable. The absence of these data may limit the generalizability of the findings across diverse populations. Sample sizes and experimental group allocations are specified in the corresponding figure legends.

PBMCs were isolated using a blood Ficoll paque (GE Healthcare, cat#17-1440-02) gradient. Blood was diluted with prewarmed PBS(Gibco, cat#10010-023) (supplemented with 0.4% of EDTA (Invitrogen, Thermofisher scientific, cat#AM9260G)). The diluted blood was layered 2:1 over Ficoll paque and centrifuged at 400 x G, 30 min at room temperature. PBMCs were extracted from the interface between Ficoll and washed and resuspended with more staining buffer for viability counts. The appropriate cell count was then used for blood DC enrichment. PBMCs were resuspended to 80 μl per 100 million cells in staining buffer in preparation for depletion of lineage cells: T cell (CD3^+^) (Miltenyi Biotec, cat#130-097-043), B cells (CD19^+^) (Miltenyi Biotec, cat#130-119-475), monocytes (CD14^+^) (Miltenyi Biotec, cat#130-050-201), NK (CD56^+^) (Miltenyi Biotec cat#130-097-042), and granulocytes(Miltenyi Biotec, cat#130-126-448) using Miltenyi positive isolation beads. The protocol for positive selection was used according to manufacturer’s protocol (Miltenyi). The negative flow through was then further negatively enriched for blood DCs (Invitrogen, cat#11308D) using manufacturer’s protocol to yield DCs which contained CD1c^+^, CD141^+^ and pDC.

#### Cell culture

Virus specific T cell generation: VST media is 1:1 ratio of RPMI1640 (Gibco, ThermoFisher, 11875093) and Click medium (FUJIFILM Irvine scientific Inc., 9195) supplemented with 10% FBS Hyclone, GE Healthcare, SH30071.01), and 2 mM L-glutamine (Gibco, ThermoFisher, 35050061).

At day 0, PBMCs from these donors were obtained from a pool of blood donors that have previously been HLA-Typed from (BGA analysis). The PBMCs were then depleted of CD56^+^ cells (To remove NK-T and NK cells) using positive isolation (Miltenyi Biotec, 130-050-401) and cells were resuspended to 1 million cells/ml. 100 μl of this solution was seeded in a 96 well U-bottom plate and 2X concentration of cytokines 1000 IU/mL GM-CSF (Miltenyi Biotec, 130-093-868), 500 IU/mL IL-4 (Miltenyi Biotec, 130-093-924) and 50 ng/mL Flt3-L (Miltenyi Biotec, 130-096-480) was added and incubated for 24 h at 37°C.

At day 1, 100 μL of media was removed and replaced with 2X concentration of 10 μm R848 (Invivogen, tlrl-r848-5), 100 ng/mL LPS (2254154, Invitrogen, 00-4976-93), 10 ng/mL IL1-β (Miltenyi Biotec, 130-093-898) and 0.1 μg/mL of each Influenza A/04/09/California peptide mix (JPT technologies, PM-INFA-HACal) peptide for 24 h at 37°C. At Day 2, 5 and 7, 100 μL of media was removed and replaced with 2X concentration of 10 IU/mL IL-2 (Miltenyi Biotec 130-097-744), 10 ng/mL IL-7 (Miltenyi Biotec 130-095-362) and 10 ng/mL IL-15 (Miltenyi Biotec, 130-095-766) and incubated at 37°C. At day 9, autologous monocyte derived DCs (moDCs) previously generated (refer to moDC generation) and pulsed with 100 μL of 1 ng/μL each Influenza A/04/09/California peptide mix (JPT technologies, PM-INFA-HACal) for 45 min at 37°C. Autologous moDCs were then added in a ratio of 1:20 (moDC: VST) and cultured in a Grex-24 plate, with 10 ng/mL IL-7 and 100 ng/mL IL-15 and incubated at 37°C. At day 11 and 13, 10 IU/mL IL-2 was added to each G-Rex 24 well (Wilson wolf, 80192M). The HAVSTs were then harvested at day 15 of culture.

The VSTs were then functionally tested to ensure that they produce IFN-γ during a restimulation of peptide mix. Briefly, 0.2 million HAVSTs were added to each well and cultured with either 50 ng/mL PMA (Sigma, 1585) and 1 μg/mL ionomycin (Sigma, I0634), 1 ng/μL each Influenza A/04/09/California peptide mix (JPT technologies, PM-INFA-HACal) or 1% Dimethlysulfoxide (DMSO) (Sigma, D2650-100 ML) for 24 h and incubated at 37°C. The supernatants were harvested and tested for their concentration of soluble IFN-γ during a restimulation of peptide mix.

### Method details

#### Viral infection

Viral stocks of RSV (ATCC, cat# VR-1540P) and Influenza (A/PR/8/34) (Charles river, cat# 10100374) were initially titrated and diluted to MOI5 and 20 ng/ml respectively using 2% FBS (Hyclone, GE Healthcare, cat# SH30071.01) in cRPMI media. Isolated blood DCs were seeded as 0.5 × 10^6^ per well. Thereafter, diluted viral stocks was added to treated wells with other treatments e.g., 25 μg/ml Poly I:C (Miltenyi Biotec, cat# 130-112-563) and 1 μg/ml lipopolysaccharide (LPS) (Invitrogen, Life technologies, cat# 00-4976-93) (stocks diluted with Hanks balanced salt solution (HBSS) (Gibco, cat# 14175095). Appropriate volumes of HBSS were also added to untreated control wells. The blood DCs were then incubated with virus for 18 h and harvested for flow cytometry staining. In other experiments where complete Roswell Park Memorial Institute (cRPMI) media is used, the formulation is as such RPMI 1640 (Gibco, ThermoFisher, cat# 11875093), 10% FBS (Hyclone, GE Healthcare, cat# SH30071.01), 100 U/ml penicillin and 100 μg/mL streptomycin (Gibco, cat# 15140122), 1 mM sodium pyruvate (Gibco, ThermoFisher, cat# 11360070), 2 mM L-glutamine (Gibco, cat# 35050061), 1x nonessential amino acids (Gibco, cat# 11140050), 15 mM HEPES (Gibco, cat# 15630080). FBS concentrations may vary and is explicitly stated.

#### Flow cytometry

Prior to flow cytometry staining of any panel and experiment, the plates housing the blood DCs were first pelleted through centrifugation at 400 X G, 5mins at 4°C and supernatants were harvested and snap frozen at −80°C. The cells are then washed once with staining buffer and pelleted through centrifugation at 400 X G, 5mins at 4°C before commencing the flow cytometry staining protocols documented below.

Blood DCs were first stained with a cocktail containing Near IR live/dead viability stain (1868118, Invitrogen, Thermofisher scientific, L34976) 1:1000, supplemented with fc receptor blocking antibody (FCR) 1:10 (2235896, Invitrogen, eBioscience, 14-9161-73) for 10 min at 4°C. The cells were washed and resuspended with cell surface marker cocktail containing CD3 (clone UCH1, BD, PECF594, 562280), CD19 (clone HIB19, BD, PECF594, 562294), CD14 (2255936, Life Technologies, PECy5.5 MHCD1418), CD56 (clone NCAM16.2, BD, PECF594, 564849), CD16 (clone 3G8, BD, BUV496, 564653), CD11c (clone B-Ly6, BD, BUV661, 612967), CD123 (clone 9F5, PECy5, BD, 551065), BDCA4 (clone Neuropilin U21-1283, BD BV605, 743130), CD1c (clone F10/21A3, BD, BUV805, 748722), CD141 (clone 1A4, BD, BV711, 563165), HLA-DR (clone G46-6, BD, V500, 561224), CD80 (clone l307.4, BD, BB790, 624296), CD83 (clone HB15e, BD, BUV786, 565336), CD86 (clone FUN-1, BD, BUV737, 612784), ILT3 (clone ZM4.1, Biolegend, PECy7, 333012), PD-L1 (clone MIH1, BD, BV421, 563738), CLIP (clone Cer.CLIP, BD, BUV395, 742601), CD40 (clone 5C3, BD Alexa Fluor 700, 561208), and intracellular markers HLA-DOβ (clone DOB.L1, Santa Cruz biotechnology, Alexa Fluor 546, sc-69739), HLA-DM (clone REA406, Miltenyi Biotec, APC, 130-124-25), RSV (clone I25J, Invitrogen, FITC, MA1-7289), FLU (clone D67J, Abcam, FITC, ab210526) for 30 min at 4°C. For inhibitor experiments that included MARCH E3 ligases, the flow cytometry panel was modified with the following markers CD3 (clone SK7, BD, APCCy7, 561800), CD19 (clone SJ25C1, BD, APCCy7, 557791), CD56 (clone NCAM.1, BD, APCCy7, 557747), CD14 (clone MΦP-9, BD, BV711, 563372), CD141 (clone 1A4, BD, BB630, 624294), ILT3 (clone ZM3.8, BD, BV750, 747371), CD45 (clone 2D1, BD, BUV563, 624284), and intracellular markers MARCH8 (polyclonal, Thermofisher scientific, PA5-20632), MARCH1 polyclonal, Thermofisher scientific, PA5-144822), MARCH9 (clone 2B5, Thermofisher scientific, H00092979-M01). MARCH8, 1 and 9 antibodies were conjugated using PECy5 conjugation kit- Lightning link (Abcam, ab102893), PE/Texas Red conjugation kit- Lightning link (Abcam, ab269899) and PECy7 conjugation kit- Lightning link (Abcam, ab102903) respectively. The cells were then washed and resuspended with fixative (FOXP3/Transcription staining buffer set, Invitrogen, 00-5523-00) for 20 min at room temperature. The cells were washed and resuspended with perm wash buffer (FOXP3/Transcription staining buffer set, Invitrogen, 00-5523-00). The cells were incubated with FCR for 30 min at 4°C. The cells were washed and resuspended with intracellular antibody cocktail for 30 min at 4°C. The cells were then washed and resuspended in staining buffer (10% Fetal calf serum, 0.4% EDTA diluted in PBS (Gibco, 10010-023)) and acquired on the BD symphony flow cytometer. Flow cytometry output was exported from Flowjo v6.0 and plotted on Prism (GraphPad). Flow cytometry data is gated based on time series, singlet exclusions based on SSC-A/SSC-H and FSC-A/FSC-H, Side scatter (SSC-A) and Forward Side scatter (FSC-A) profile exclusion. The cells were then further gated based on live cells and the phenotype of CD1c^+^ DCs identified in the full gating strategy.

#### Confocal imaging

Prior to confocal imaging analysis, a cell carrier-384 ultra plate (384 PerkinElmer, 6057300) is coated with 30 μL of water solubilized Poly-D-lysine hydrobromide (Sigma, P7886). The plate is incubated for 1 h at room temperature to allow the Poly-D-Lysine to coat the well. The wells are then washed with 50 μL sterile water (Thermofisher scientific, 15230170) twice before drying in a biosafety cabinet (BSC) hood. Once dry, the plate is then covered until cells are ready to be imaged. This step allows stained cells to adhere onto the plate in a monolayer before imaging.

Positively enriched CD1c^+^ DCs (Miltenyi Biotec, 130-119-475) were plated and infected with RSV, FLU and Poly I:C (Miltenyi Biotec, 130-112-563) separately in the same way the cells were evaluated for flow cytometry. Post 18-h incubation, the cells were harvested and fixed immediately with 4% para formaldehyde (Alfa Aesar, 47377) and incubated at room temperature for 20 min in the dark. The cells were then washed with PBS (Gibco, 10010-023) supplemented with 0.1% Triton X-100 (Biobasic, TB0198) (PBST) and blocked with 5% BSA for 1 h at room temperature. The cells were then resuspended with an antibody cocktail mix consisting of HLA-DR (clone L243, Miltenyi Biotec, AF647, ab239277), HLA-DM (clone REA406, Miltenyi Biotec, PE, 130-122-913) and LAMP1 (clone H4A3, Abcam, Alexa flour 488, ab187591) and incubated for 45 min at room temperature. Post incubation, the cells were washed with PBST by centrifugation at 400 X G, 5 min at 4°C and resuspended with nuclear dye, 1 μM Hoechst Trihydrochloride, Trihydrate (Thermofisher, 33342) dissolved in PBS (Gibco, 10010-023) for 10 min at room temperature.

For experiments on IRF3 nuclear localization, positively enriched CD1c^+^ DCs were stimulated with PIC with or without BX795, together with RSV infection for 8 h. The cells were then fixed and permeabilized as documented above and stained with HLA-DR (clone L243, Miltenyi Biotec, AF647, ab239277), IRF3 (cloneD9J5Q, Cell Signaling Technologies, AF488, 67651S) and p65 (cloneD14E12, Cell signaling technologies, unconjugated, 8242S) overnight. Following overnight incubation, the cells were washed with staining buffer and resuspended with secondary antibody goat anti-Rabbit IgG H&L (Abcam, Alexa Flour 555, ab150078) for 30 min at room temperature.

The cells were then washed and seeded onto a pre coated poly-L-lysine cell carrier-384 ultra plate (384 PerkinElmer, 6057300). The 384 well plate is then quickly spun at 300X G for 1 min at room temperature to allow cells to settle and adhere to the bottom of the plate. The cells were then imaged on the Zeiss LSM800 inverted microscope using the 63X oil immersion objective (Plan-Apochromat 63×/1.40 Oil DIC M27). The acquired images were then imported into the Zen Blue software (ZEISS) for image analysis, where histograms are equal between all images and exported from the same software. The scale bars attached to each image is 5 microns in length.

#### SMK/Abseq staining

Abseq antibodies and Sample Multiplexing Kit (SMK) reagents including a custom Abseq reagent (anti-CD304, clone U21-1283) was obtained from BD. Staining protocols for the Abseq + SMK were as per manufacturer’s protocol (Doc ID: 214419 Rev. 2.0), except for the following minor change: The concentration of each Abseq oligo used in the experiment was halved (1.0 mL). Following Abseq + SMK staining, cells were then resuspended in staining buffer and viability count was performed.

#### scRNA library preparation and sequencing

At each timepoint, a separate BD Rhapsody cartridge was used. For each cartridge, Abseq + SMK-stained cells from each donor and condition were pooled in equal numbers before processing via the BD Rhapsody Express system. A total count of 40,000 cells was attempted for each cartridge. Library preparation was performed as per manufacturer’s protocols (Doc ID: 23–24120 Rev. 1.0). Final sequencing libraries were pooled and sequenced on a single lane of Novaseq X, together with 20% PhiX spike in as per BD’s recommendations.

Sequence data QC and analysis was performed using R (version 4.2.2) and Seurat (version 4.3.0). Briefly, initial single cell QC was performed for samples from each timepoint by removing SMK multiplets, dead cells by percentage mitochondrial reads, and doublets by both count and feature depth. Single cells that passed initial QC were then utilized for Seurat’s integration workflow. All samples at T = 4, 8 and 18 h detected Abseq staining, except that the 0 h timepoint showed poor Abseq staining across all DC subsets. Hence, CD1c^+^ DC cell clusters at 0h were identified in the integrated dataset via both their average staining for CD1c^+^ Abseq antibody, in combination with their negativity for the following canonical markers for other cell types: MS4A1, FCGR3A, FCER1A, and CD304(Abseq).

Differential gene and Gene pathway analysis between clusters was performed using Seurat’s embedded MAST^134^ and DEenrichR^87-89^ algorithms, on genes meeting the *p*-value cutoff (*p* < 0.05) as measured by the MAST statistical test and GO_Biological_Process_2021 as the selected reference database.

#### Inhibitor assays

Inhibitors used in this experiment include 1 μM BX795 (Axon Medchem, 1390), MRT 67307 (Med Chem Express, HY-13018∼10 mg), 1 μM Tofacitinib citrate (Tocris, 4556). The inhibitors were used in the context of activation using 25 μg/ml Poly I:C (Miltenyi Biotec, 130-112-563) or 1 μg/ml each of RIG-I/MDA5 LMW (Invivogen tlrl-picwlv) and HMW (Invivogen tlrl-piclv). Inhibitors were incubated with blood DCs for 30 min prior to adding Poly I:C for 18 h. The cells were then stained, acquired using Flow cytometry and the output was analyzed on Flowjo v6.0 and plotted on Prism (GraphPad version 9.5.1).

#### Phosphorylation-flow cytometry

Blood DCs were stimulated with Poly I:C (Miltenyi Biotec, 130-112-563) and other drugs and incubated for 2 h at 37°C. The cells were then immediately stained with a live/dead viability dye at room temperature. The cells were immediately pelleted by centrifugation at 3000 x RPM, 30 s at 4°C and immediately fixed with prewarmed BD phosfix buffer 1(BD, 557870) and incubated at 37°C. The cells were then washed and permeabilized with BD phosflow permwash buffer 1 (BD, 557885). The cells were then stained overnight with antibody markers CD11c (B-Ly6, BD, BUV661, 612967), Lin1 (BD, FITC, 340546), CD1c (clone F10/21A3, BD, BUV805, 748722), CD56 (clone B159, BD, Alexa Fluor 700, 557919), CD123 (clone 9F5, PECy5, BD, 551065), BDCA4 (clone Neuropilin U21-1283, BD BV605, 743130), *p*-IRF3 (Ser396) (clone D601M, Cell Signaling Technologies, 83611S) overnight at 4°C in the dark. A portion of cells were taken and incubated for a total of 24 h, where we then evaluate the expression of total IRF3 using these antibody markers CD3 (clone UCH1, BD, PECF594, 562280), CD19 (clone HIB19, BD, PECF594, 562294), CD56 (NCAM16.2, BD, PECF594, 564849), CD16 (clone 3G8, BD, BUV496, 564653), CD11c (clone b-Ly6, BD, BUV661, 612967), CD123 (clone 9F5, PECy5, BD, 551065), BDCA4 (clone Neuropilin U21-1283, BD BV605, 743130), CD1c (clone F10/21A3, BD, BUV805, 748722), CD45 (clone 2D1, BD, BUV563, 624284), HLA-DR (clone G46-6, BD, V500, 561224), CD80 (clone l307.4, BD, BB790, 624296), CD86 (clone FUN-1, BD, BUV737, 612784), CD14 (clone MΦP-9, BD, BV711, 563372). All cells were then acquired on the BD FACSymphony.

#### Chemokine/cytokine analysis

Cellular supernatants previously frozen are thawed on ice prior to measurements. These supernatants represent the soluble cytokine and chemokines that were secreted by cells during stimulation or infection. Cell culture supernatants were measured using Milliplex 9-plex Human Interferon Panel (Merck Millipore, HIFN-130K-09) and according to manufacturer’s protocol. This technique enables the detection of human IFNα2, IFNβ, IFNγ, IL-28B/IFNλ3, IL-28A, IL-29, IFNω, IFNε, IFNGR1. Plates were read and analyzed using the Flexmap 3D (Luminex Corp) and Bio-plex Manager software (Bio-Rad, Version 6.2).

#### Dendritic cell- viral specific T cell (DC-VST) coculture

PBMCs from autologous donors were positively enriched for CD1c^+^ DCs (Miltenyi Biotec, 130-119-475) and cultured with full length HA peptide (Influenza A/04/09/California) (Sino Biological, 11085-V08B-100) for 18 h, in the presence of 25 μg/mL Poly I:C (Miltenyi Biotec, 130-112-563), 10 μg/mL MHCI (HLA-A, B, C) blocking antibody (clone W6/32, Biolegend, 311412) to restrict T cell response to MHCII presented peptides, with and without 1 μM BX795 (Axon Medchem, 1390). Autologous HA-VSTs were also thawed and rested overnight separately in 2% FBS (Hyclone, GE Healthcare, SH30071.01) cRPMI. Post 18 h incubation, the CD1c^+^ DCs were washed thoroughly to remove Poly I:C (Miltenyi Biotec, 130-112-563) and drug traces. HA-VSTs were also stained with cell trace violet (CTV) (Invitrogen, C34557) using manufacturer’s protocol. The CD1c^+^ DCs were then added to CTV stained autologous HA-VSTs in a ratio of 1:20 (DC:T). The DC-T cell coculture was incubated for 5 days after before flow cytometry analysis. The markers used for flow cytometry staining were CD3 (clone SK7, BD, BB700, 566575), CD4 (clone SK7, BD, BV480, 566165), CD8 (clone SK1, BD, BUV805, 612889), CD45RA (clone HI100, Biolegend, 304138), CCR7 (clone 3D12, BD, PECy7, 557648), CD25 (clone 2A3, BD, BUV395, 564034), PD-1 (clone EH12.1, BD, BUV737, 612791), CD69 (clone FN50, BD, BUV563, 748764), CD56 (BB630), CD11c (clone B-Ly6, BD, BUV661, 612967), KLRG1 (clone Z7-205.rMab, BD, PECF594, 568658), CD95 (DX2, Biolegend, Alexa Fluor 700, 305647), CD39 (clone Tu66, BD, BV605, 742522), CD73 (clone AD2, BD, BV786, 742635), CD57 (clone NK-1, BD, BUV615, custom conjugated), FOXP3 (clone 259D/C7, BD, PE, 560046),Ki67 (clone Ki67, Biolegend, Alexa Fluor 488, 350508) and CTLA4 (clone BNI3, BD, PECy5, 555854).

### Quantification and statistical analysis

Statistical analysis was performed using Prism (GraphPad, version 9.5.1). Datasets were compared using non-parametric Friedman statistical calculations, unless otherwise stated. Data are presented as median ±standard error of mean (SEM). *p* values were reported in values, and *p* > 0.9999 were not shown.
