# Supplementary material for: Respiratory syncytial virus inhibits type I interferon signaling to maintain HLA-DM expression in CD1c+ dendritic cells
Source: iScience. 2026 Jul 10;29(8):116736. doi: 10.1016/j.isci.2026.116736 (PMC13380456; doi:10.1016/j.isci.2026.116736)
Supplement: Document S1. Figures S1–S5 and Table S1 [file mmc1.pdf]

iScience, Volume *29*

Supplemental information

Respiratory syncytial virus inhibits type I interferon signaling to maintain HLA-DM expression in $CD1c^+$ dendritic cells

Weiyee Ong, Richard Anthony Hopkins, Enjun Yang, Novita, Najwa Talib, Bijin Au, and John Edward Connolly

Supplementary figures

Supplemental information

Document S1. Figures S1-S5, Table S1

Supplementary Figure 1

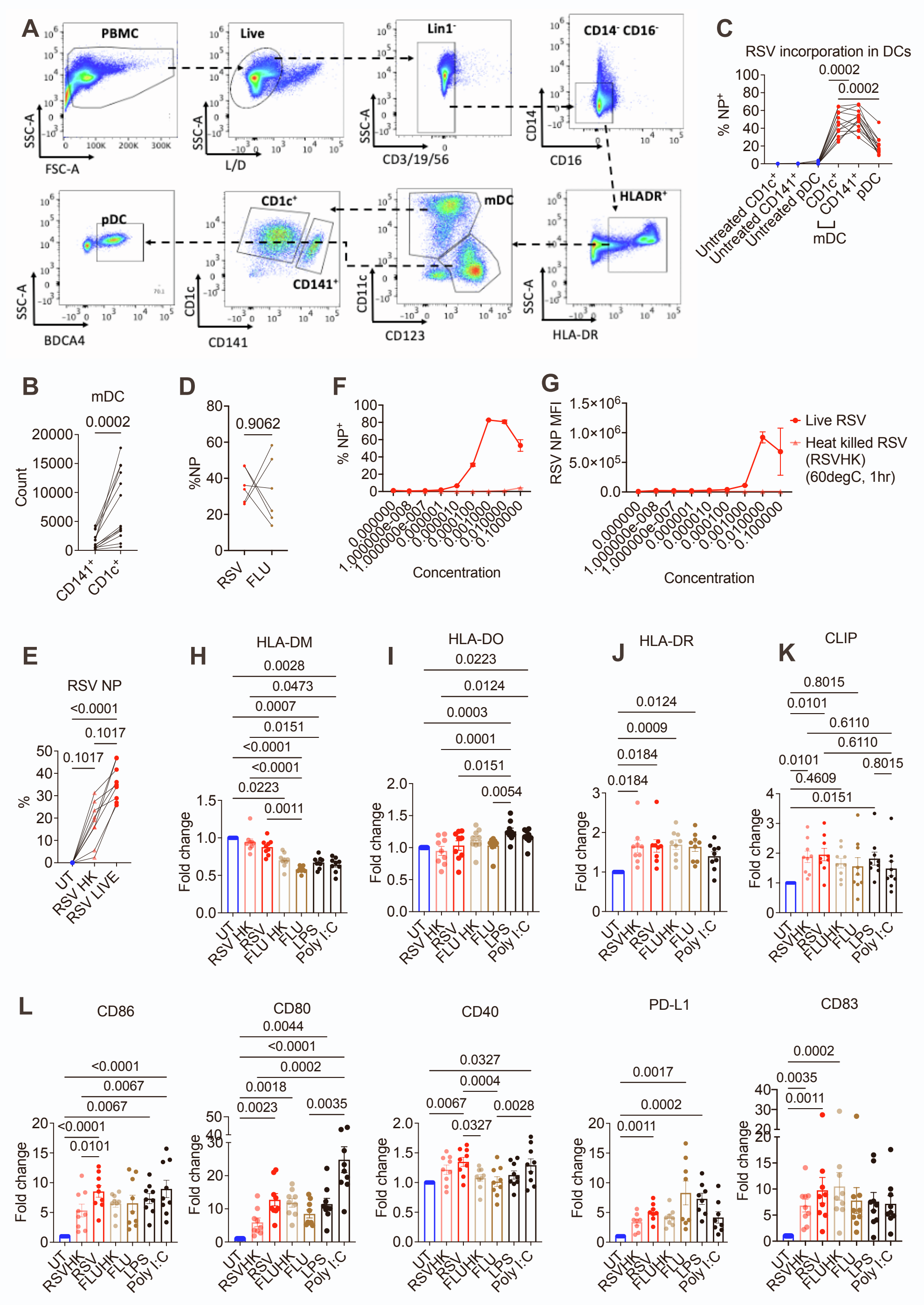

Supplementary figure 1. RSV maintains HLA-DM in CD1c$^+$ DCs compared to FLU, LPS and Poly I:C (PIC) treatments, related to Figure 1B-D

Blood dendritic cells were infected with RSV and FLU for 18 hours, median ± SEM

(A) RSV-NP$^+$ frequencies in dendritic cell subsets. The percentages of RSV-NP$^+$ cells within CD1c$^+$ and CD141$^+$of the myeloid dendritic cell subset (mDCs) and plasmacytoid dendritic cells (pDCs) are shown as a paired dot plot, with each pair of points representing values from the same donor. Wilcoxon matched-pairs signed-rank test was used, N=13.

(B) Flow cytometry gating strategy of enriched blood DCs from PBMCs. Dashed arrows represent drill down of the selected gate for further characterization of cells. Side scatter (SSC-A) and forward side scatter (FSC-A) determine PBMCs. Live cells are selected from live/dead (L/D) negative population (Near IR, Invitrogen). Major cell lineages (Lin1), based on the expression of CD3/19/56 were used to depict T , B and NK cells respectively. Monocytes and certain DC populations of monocytic origin were determined by CD14 and CD16 expression. Blood DCs are gated based on their expression of MHCII molecule HLA-DR. mDCs (CD1c$^+$ and CD141$^+$ DCs) are gated out based on their expression for CD11c$^+$ and further characterized into CD1c$^+$ and CD141$^+$ subsets. pDCs are selected based on their expression of lineage markers CD123 and BDCA4 expression.

(C) Flowjo cell count output of CD1c$^+$ and CD141$^+$ mDCs

(D) Percentage (%) RSV nucleoprotein (RSV-NP$^+$) or FLU nucleoprotein (FLU-NP$^+$) in HEp-2 cells. NP$^+$ cells were gated on mock infection controls and percentage NP$^+$ plotted between paired donors, N=6.

(E) RSV-NP$^+$ of RSVHK and RSV infected CD1c$^+$ DCs, T = 18h N=8

(F-G) Percentage and MFI of total RSV-NP$^+$ between RSV-HK and live RSV infected HEp-2 cell line, T = 18 h, N=3

(H-L) Fold changes of HLA-DM, HLADOβ, HLA-DR, CLIP, CD86, CD80, CD40, PD-L1 and CD83 of RSVHK, RSV, FLUHK, FLU, lipopolysaccharide (LPS) and PIC infected CD1c$^+$ DC T = 18h. Fold changes were calculated based on corresponding MFI of untreated cells

Supplementary Figure 2

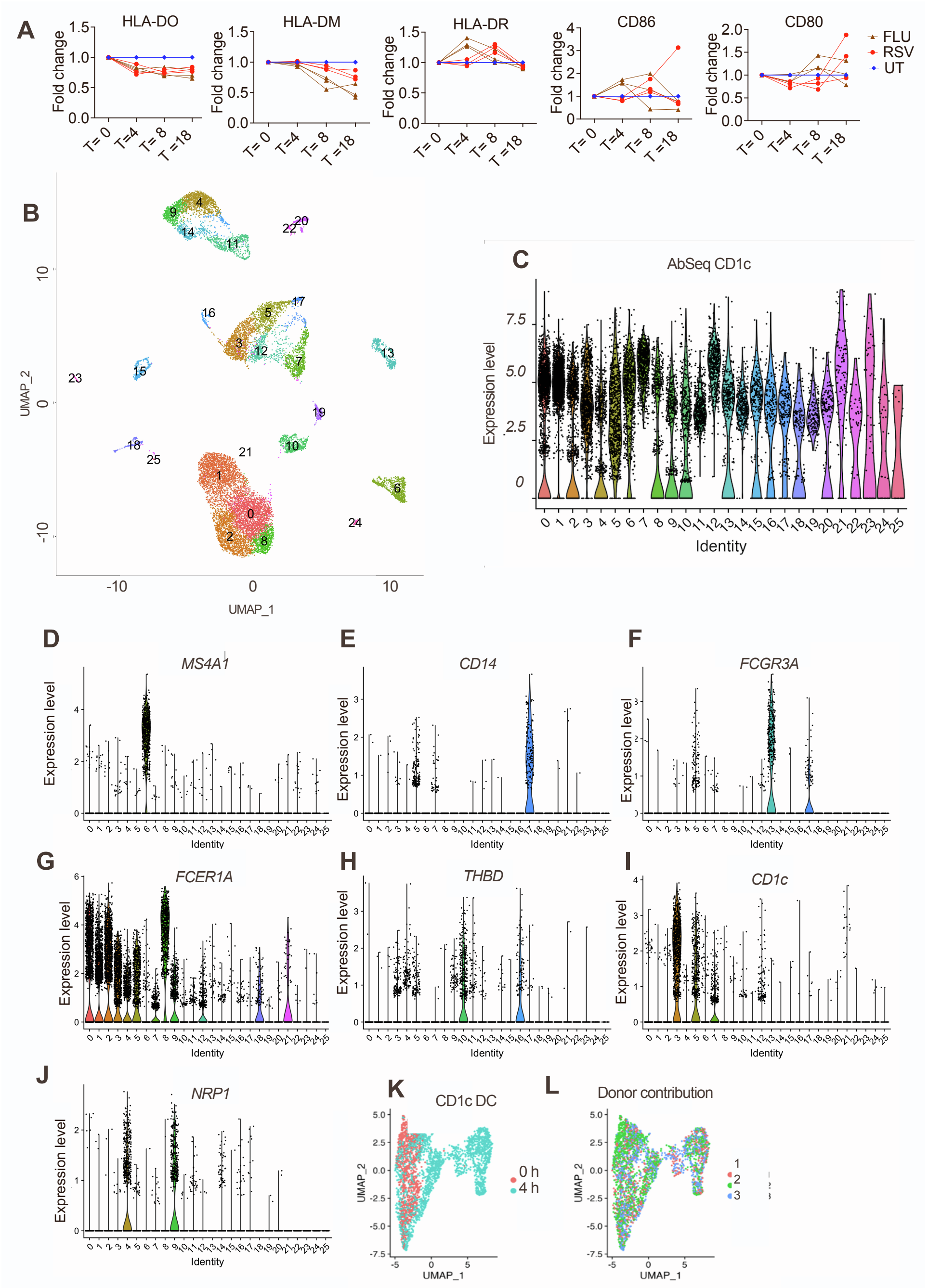

Supplementary figure 2. Type I interferon (IFN) pathway attenuation in CD1c^{+} DCs during RSV infection, Related to Figure 2A

(A) Flow cytometry MFI HLA-DR, CD86 and CD80, HLA-DO and HLA-DM from CD1c^{+} DCs of the three donors used for scRNA sequencing, N= 3

(B) UMAP clustering of all sequenced cells based on RNA expression

(C) Abseq oligo tag protein expression of CD1c based on cluster classifiers in B

(D-J) RNA expression of *MS4A1*(CD19), *CD14*, *FCGR3A* (CD16), *FCER1A*, *THBD* (CD141) and CD1c based on their cluster classifiers in B

(K-L) UMAP of RSV infected CD1c^{+} DCs after T= 4 h (Teal) overlayed against T= 0 h uninfected CD1c^{+} DCs (Red) and relative donor distribution, N= 3

Supplementary Figure 3

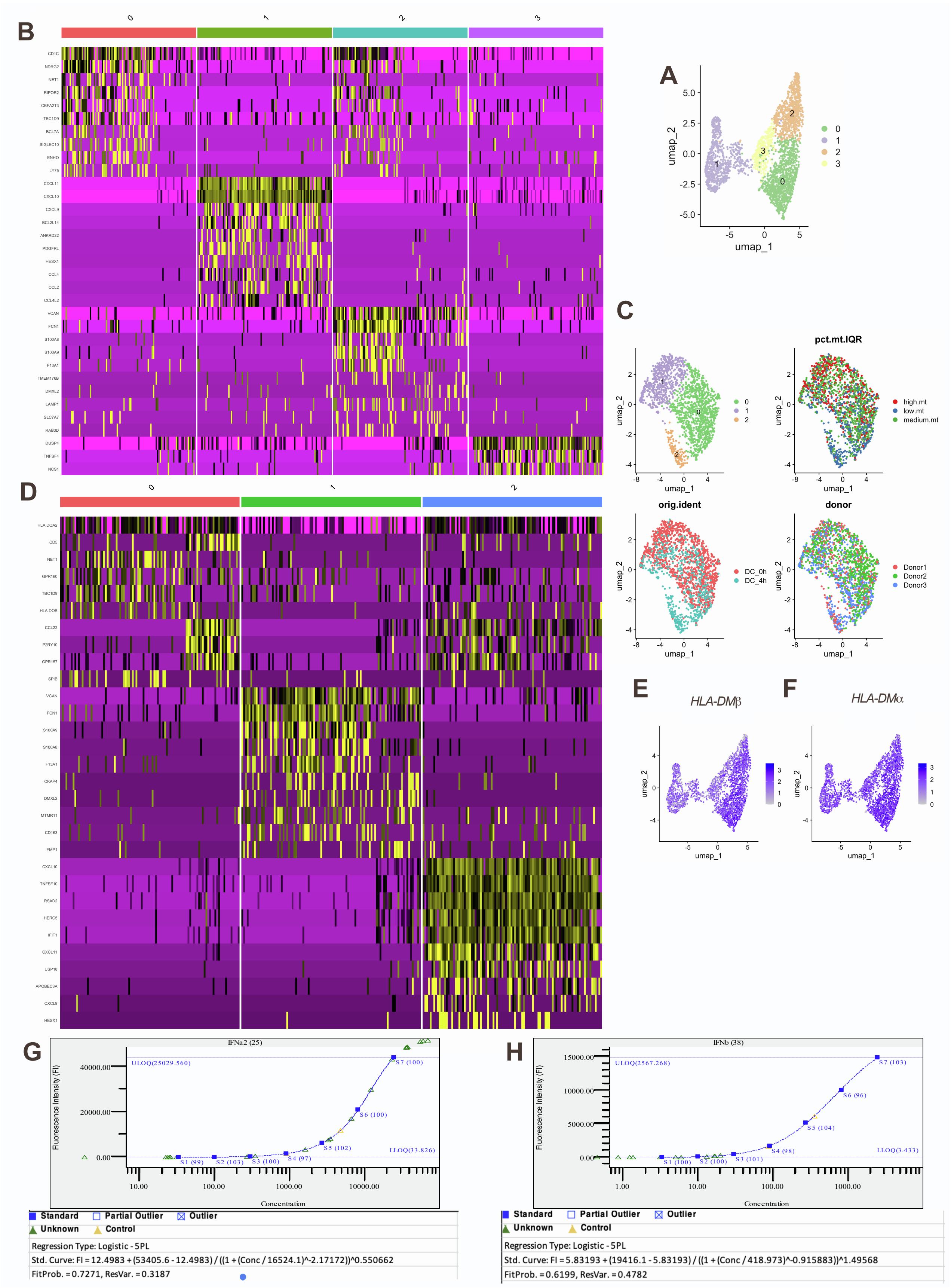

Supplementary Figure 3. Additional clustering analyses of scRNA sequenced RSV and FLU infected CD1c+ DCs, Related to Figure 2

(A-B) Reclustering of all CD1c^{+} DCs (untreated, RSV, and FLU) from the 0 h and 4 h timepoints at increased resolution, identifying four transcriptionally distinct subclusters. UMAP coloured by cluster identity (0-3); Cluster 0: classical CD1c^{+} DCs; Cluster 1: IFN-stimulated DCs; Cluster 2: CD14^{+} monocyte-associated DCs; Cluster 3: maturation-associated DCs. Heatmap of the top 25 differentially expressed genes per cluster (ranked by log2 fold change) is shown alongside. N = 3 donors.
(C-D) Focused reclustering of untreated CD1c^{+} DCs only, resolving three subpopulations. Top left: UMAP coloured by cluster identity (0-2); top right: UMAP coloured by mitochondrial RNA percentage (IQR-based classification: low, medium, high), ; bottom left: UMAP coloured by timepoint; bottom right: UMAP coloured by donor. Cluster 0: classical cDC2 (enriched for HLA-DQA1, HLA-DQA2, HLA-DOB); Cluster 1: CD14^{+} inflammatory DC subset (VCAN, FCN1, S100A8/A9); Cluster 2: basally IFN-primed cells (MX1, CXCL10, IFIT1/3). Heatmap of the top 25 differentially expressed genes per cluster (ranked by log2 fold change) is shown alongside. N = 3 donors.
(E-F) Heatmap visualization of HLA-DMβ and HLA-DMα on the UMAP (Figure 2A) at 4h post infection
(G-H) Standard curves for IFNα and IFNβ and their respective standard curve fits from a 5PL model. Data was exported from Luminex supernatant readout of IFNα and IFNβ of CD1c^{+} DCs used for scRNA sequencing experiments, N = 3, see Table S1

Supplementary Figure 4

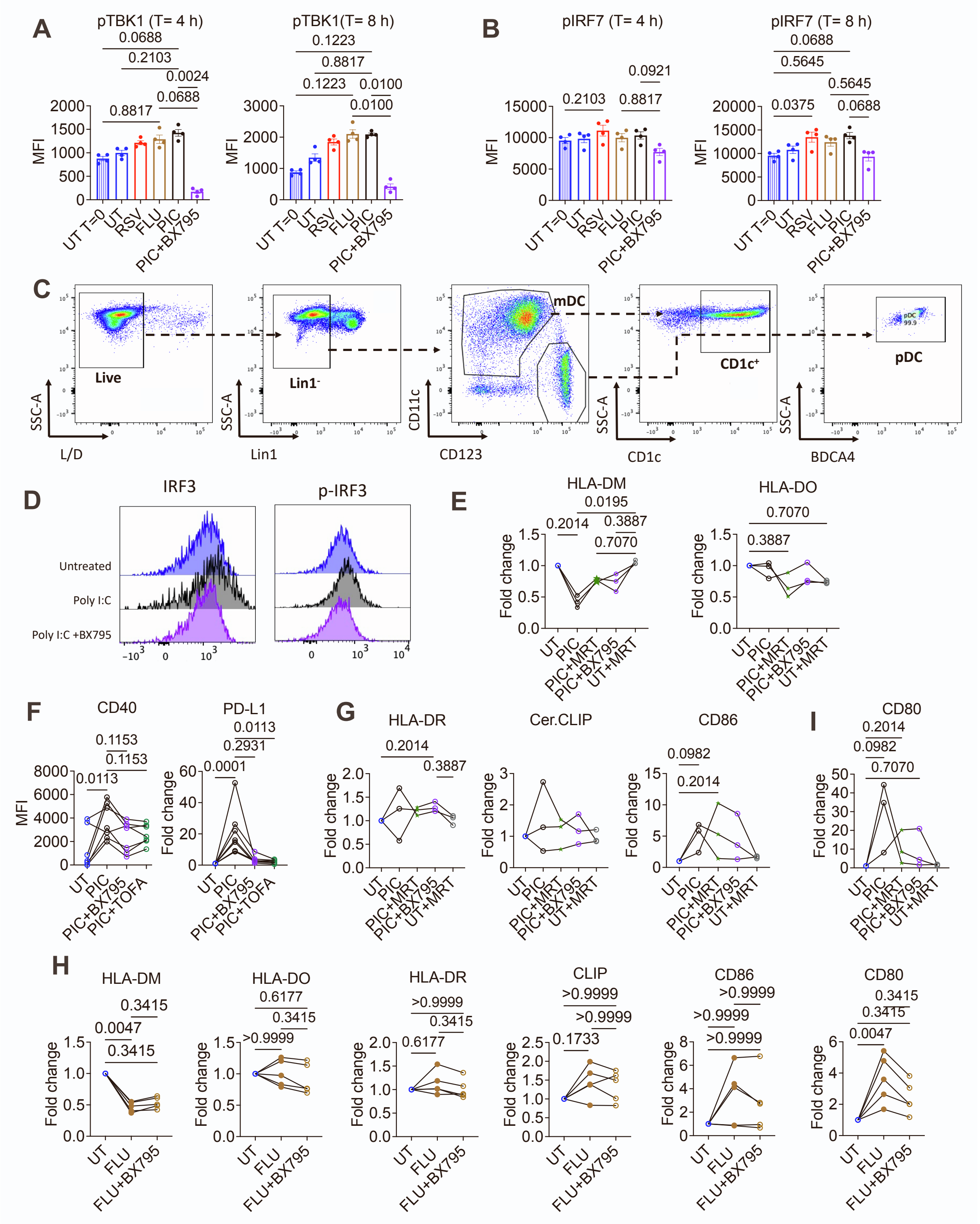

Supplementary figure 4. TBK1 associated HLA-DM regulation during DC activation, Related to Figure 3A, C-D,G)

(A-B) MFI of phosphorylated TBK1 and IRF7 of CD1c⁺ DCs either infected with RSV, FLU or PIC with or without BX795 compared against untreated cells after T= 0, 4 and 8 h, median ± SEM.

(C) Flowjo gating strategy CD1c⁺ DCs from enriched blood DCs

(D) Flowjo histogram of IRF3 and p-IRF3 expression in CD1c⁺ DCs stimulated with PIC, with or without BX795, of a representative donor at T= 2 h.

(E) Fold change of HLA-DM, HLA-DO, of CD1c$^+$ DCs stimulated with PIC, with or without BX-795/MRT67307 (MRT), N= 3, median ± SEM. Statistical comparisons not annotated are P>0.9999.

(F) MFI and Fold change of CD40 and PD-L1 expression respectively of CD1c$^+$ DCs stimulated with PIC, with or without BX795, N = 7, median ± SEM

(G) Fold change of HLA-DR, cer.CLIP, CD86 and CD80 expression of CD1c$^+$ DCs stimulated with PIC, with or without MRT67307 (MRT) or BX795 from (G), N= 3, median ± SEM

(H) Fold change of HLA-DM and HLA-DO, HLA-DR, CLIP, CD86 and CD80 of CD1c$^+$ DCs stimulated with FLU, with or without BX795, N= 5

Supplementary Figure 5

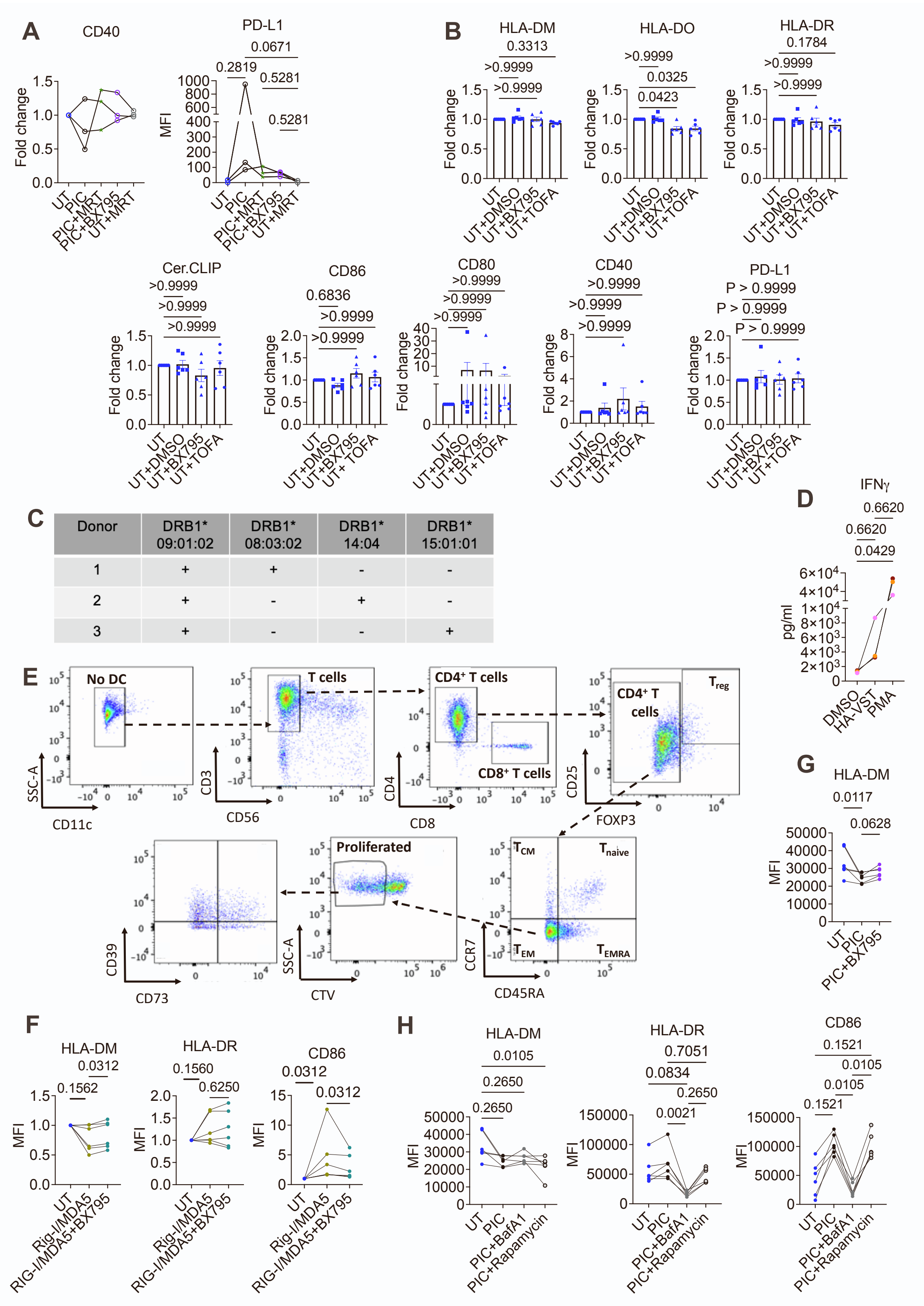

Donor	DRB1* 09:01:02	DRB1* 08:03:02	DRB1* 14:04	DRB1* 15:01:01
1	+	+	-	-
2	+	-	+	-
3	+	-	-	+

Supplementary figure 5. HLA-DM^{hi} DCs effect on HA primed VST$_{em}$ models a HAVST$_{em}$ phenotype of altered function, Related to Figure 4A.

(A) MFI and Fold change of CD40 and PD-L1 expression respectively of CD1c$^+$ DCs stimulated with PIC, with or without MRT67307 (MRT) or BX795 from (G), N = 3, median ± SEM
(B) Fold change of HLA-DM, HLA-DO, HLA-DR, Cer.CLIP, CD86, CD80, CD40 and PD-L1 of CD1c$^+$ DCs cultured with 1% Dimethlysulfoxide (DMSO), BX795 or TOFA separately for T= 18 h, N= 6, median ± SEM
(C) Specific HLA-DR alleles of three selected donors.
(D) Flowjo gating strategy of HAVST$_{em}$.
(E) Supernatant analysis of IFNγ (pg/ml) from HA-VST$_{em}$ during repriming with either DMSO, Influenza HA peptide (HA) or PMA for T= 18 h, N= 3, median ± SEM
(F) MFI of HLA-DM, HLA-DR and CD86, of CD1c$^+$ DCs stimulated with RIG-I/MDA5, with or without BX795, N= 6. Wilcoxon matched pairs signed rank test used for statistical analysis, median ± SEM
(G) MFI of HLA-DM expression of PIC with and without BX795, of donors in (S5B) used to test Bafilomycin A1 and Rapamycin treatment on PIC CD1c$^+$ DCs, N= 6, median ± SEM. Statistical comparisons P >0.9999 are not shown
(H) MFI of HLA-DM, HLA-DR and CD86 of bafilomycin A1 and rapamycin treated PIC CD1c$^+$ DCs, N= 6, median ± SEM

Supplementary Table 1

	IFNα2	IFNβ	IFNα2	IFNβ	IFNα2	IFNβ
**Description**	**FI - Bkgd**	**FI - Bkgd**	**Conc in Range**	**Conc in Range**	**Obs Conc**	**Obs Conc**
Reagent Name: Background0	8.5	13.5				
Reagent Name: Standard1	45	32.3	33.83	3.43	33.83	3.43
Reagent Name: Standard2	139.3	120.3	105.56	10.24	105.56	10.24
Reagent Name: Standard3	470.3	492.5	308.92	31.32	308.92	31.32
Reagent Name: Standard4	1657.8	1714.5	901.09	90.32	901.09	90.32
Reagent Name: Standard5	6407	5216.5	2829.41	288.11	2829.41	288.11
Reagent Name: Standard6	20988	10071.3	8304.22	802.76	8304.22	802.76
Reagent Name: Standard7	44273.5	14968	25029.56	2567.27	25029.56	2567.27
QC-1	1695.5	1675.5	918.38	88.45	918.38	88.45
QC-2	11880.5	6201	4846.9	361.08	4846.9	361.08
UT_JEC 22-14_0h	2	-1.5	OOR <	OOR <	OOR <	OOR <
UT_JEC 22-15_0h	4.5	-2.5	OOR <	OOR <	OOR <	OOR <
UT_JEC 22-16_0h	1.5	-0.5	OOR <	OOR <	OOR <	OOR <
UT_JEC 22-14_4h	5.5	-3.5	OOR <	OOR <	OOR <	OOR <
UT_JEC 22-15_4h	-1.5	1	OOR <	OOR <	OOR <	OOR <
UT_JEC 22-16_4h	8	1.5	OOR <	OOR <	OOR <	OOR <
RSV_JEC 22-14_4h	34.5	-1.5	OOR <	OOR <	*24.41	OOR <
RSV_JEC 22-15_4h	35.5	-3.5	OOR <	OOR <	*25.33	OOR <
RSV_JEC 22-16_4h	33	-0.5	OOR <	OOR <	*23.01	OOR <
Flu_JEC 22-14_4h	17274.5	1	6848.46	OOR <	6848.46	OOR <
Flu_JEC 22-15_4h	3414.5	-0.5	1658	OOR <	1658	OOR <
Flu_JEC 22-16_4h	8354.5	0.5	3555.92	OOR <	3555.92	OOR <
UT_JEC 22-14_8h	4.5	-0.5	OOR <	OOR <	OOR <	OOR <
UT_JEC 22-15_8h	1	4.5	OOR <	OOR <	OOR <	OOR <
UT_JEC 22-16_8h	5	1.5	OOR <	OOR <	OOR <	OOR <
RSV_JEC 22-14_8h	551.5	7.5	354.16	OOR <	354.16	*0.45
RSV_JEC 22-15_8h	42.5	2.5	OOR <	OOR <	*31.64	OOR <
RSV_JEC 22-16_8h	410	3	274.53	OOR <	274.53	OOR <
Flu_JEC 22-14_8h	49160.5	13.5	OOR >	OOR <	*38165.60	*1.38
Flu_JEC 22-15_8h	29960.5	1.5	12423.87	OOR <	12423.87	OOR <
Flu_JEC 22-16_8h	51476.5	29.5	OOR >	OOR <	*56554.46	*3.17

Table S1. Supernatant values taken from Luminex readout, Related to Figure S3G-H

(A) Fluorescence intensity background subtracted values (FI- Bkgd) and concentration in Range (pg/ml) of each data point is listed. Values that fall below and above the standard curve is listed as out of range (OOR<) or out of range (OOR>) respectively. Based on the OOR> or OOR< values, the Observed concentration (Obs Conc) that were extrapolated is tagged with an asterisk "*". Standards for the assay to determine the linear range is listed, while QC1 and QC2 are internal controls provided by the manufacturer.
